# Evolution of mitosome metabolism and invasion-related proteins in *Cryptosporidium*

**DOI:** 10.1186/s12864-016-3343-5

**Published:** 2016-12-08

**Authors:** Shiyou Liu, Dawn M. Roellig, Yaqiong Guo, Na Li, Michael A. Frace, Kevin Tang, Longxian Zhang, Yaoyu Feng, Lihua Xiao

**Affiliations:** 1 State Key Laboratory of Bioreactor Engineering, School of Resources and Environmental Engineering, East China University of Science and Technology, Shanghai, 200237 China; 2 Division of Foodborne, Waterborne, and Environmental Diseases, Centers for Disease Control and Prevention, Atlanta, GA 30329 USA; 3 Division of Scientific Resources, Centers for Disease Control and Prevention, Atlanta, GA 30329 USA; 4 College of Animal Science and Veterinary Medicine, Henan Agricultural University, Zhengzhou, 450002 China

**Keywords:** Reductive evolution, Genomics, Mitosome metabolism, Apicomplexa, *Cryptosporidium*

## Abstract

**Background:**

The switch from photosynthetic or predatory to parasitic life strategies by apicomplexans is accompanied with a reductive evolution of genomes and losses of metabolic capabilities. *Cryptosporidium* is an extreme example of reductive evolution among apicomplexans, with losses of both the mitosome genome and many metabolic pathways. Previous observations on reductive evolution were largely based on comparative studies of various groups of apicomplexans. In this study, we sequenced two divergent *Cryptosporidium* species and conducted a comparative genomic analysis to infer the reductive evolution of metabolic pathways and differential evolution of invasion-related proteins within the *Cryptosporidium* lineage.

**Results:**

In energy metabolism, *Cryptosporidium* species differ from each other mostly in mitosome metabolic pathways. Compared with *C. parvum* and *C. hominis*, *C. andersoni* possesses more aerobic metabolism and a conventional electron transport chain, whereas *C. ubiquitum* has further reductions in ubiquinone and polyisprenoid biosynthesis and has lost both the conventional and alternative electron transport systems. For invasion-associated proteins, similar to *C. hominis*, a reduction in the number of genes encoding secreted MEDLE and insulinase-like proteins in the subtelomeric regions of chromosomes 5 and 6 was also observed in *C. ubiquitum* and *C. andersoni*, whereas mucin-type glycoproteins are highly divergent between the gastric *C. andersoni* and intestinal *Cryptosporidium* species.

**Conclusions:**

Results of the study suggest that rapidly evolving mitosome metabolism and secreted invasion-related proteins could be involved in tissue tropism and host specificity in *Cryptosporidium* spp. The finding of progressive reduction in mitosome metabolism among *Cryptosporidium* species improves our knowledge of organelle evolution within apicomplexans.

**Electronic supplementary material:**

The online version of this article (doi:10.1186/s12864-016-3343-5) contains supplementary material, which is available to authorized users.

## Background

The evolution of life generally proceeds towards bigger genomes and increased complexity, as the organisms adapt to new niches and environment. Recent evolutionary reconstructions, however, have shown a common occurrence of genome reduction, especially in parasitic and symbiotic organisms [1]. Among alveolates, a group of unicelluar eukaryotes consisted of photosynthetic protozoa, free-living predators, and obligate intracellular parasitic protozoa, reductive evolution is often observed in parasitic apicomplexans. For example, compared with the closely related chromerids, the photosynthetic algae, a significant reduction in genome sizes has occurred in apicomplexans [2]. Among apicomplexans, *Cryptosporidium* spp. and gregarines have lost the apicoplast, a plastid without photosynthetic functions, and depend on host cells for basic nutrients [3–6]. It is generally accepted that *Cryptosporidium* spp. as the based branch of Apicomplexa have also lost many other metabolic capabilities during the reductive evolution, especially the mitochondria-like organelle-derived energy metabolism, such as the tricarboxylic acid (TCA) cycle and cytochrome-based electron transport chain [4, 5, 7]. *Cryptosporidium muris*, however, has been shown recently to have all enzymes associated with the TCA cycle and a conventional respiratory chain system [8].

*Cryptosporidium* spp. are major causes of diarrhea in human and other animals, is [9]. To date, about 30 *Cryptosporidium* species have been recognized in humans, livestock, companion animals, and wild vertebrates [10]. They differ from each other in host specificity and predilection sites [10]. Among them, *C. parvum* and *C. hominis* are intestinal species and common causes of human cryptosporidiosis [11]. Although *C. hominis* is largely a pathogen of humans and nonhuman primates, *C. parvum* is also a major pathogen in ruminants. Recently, another intestinal *Cryptosporidium* species, *C. ubiquitum*, has been detected in humans in industrialized nations [12, 13]. Like *C. parvum*, this species has a broad host range and can infect other primates, domestic and wild ruminants, and rodents [12, 13]. In contrast, *C. andersoni* is a gastric species in cattle and has only been detected occasionally in other animal species [10, 14]. It is genetically related to another gastric species, *C. muris*, which infects a broad range of mammals and occasionally birds [15]. Like *C. hominis*, most other recognized *Cryptosporidium* species have some host specificity [10].

The genomes of *C. parvum* [5] and *C. hominis* [4] were sequenced using the Sanger technology and published in 2004. *C. muris* was also sequenced subsequently and its genome has been available in GenBank and CryptoDB (release 3.5) since 2007. All *Cryptosporidium* genomes presumably have 8 chromosomes, are around 9 Mb in size, and are more compact and efficient than genomes of most other apicomplexans [4, 5]. The predicted proteomes are highly similar between the two intestinal species *C. parvum* and *C. hominis.* However, a preliminary analysis of the *C. muris* genomic data has shown significant divergence in mitosome carbon and energy metabolism [8]. Because of the overall nucleotide sequence divergence between the *C. parvum* and *C. hominis* genomes is just ~3%, it has been suggested that differences in phenotypic characteristics between the two species, such as host range [11] and host cell invasion [16], may be caused by subtle sequence variations in coding regions or differences in expression levels of key genes rather than genome rearrangements and structural alterations [17]. Recently, several major insertions and deletions in gene content have been identified between the two closely related intestinal species, and it was suggested that subtelomeric gene duplications and deletions in two secreted protein families (MEDLE and insulinase-like proteins) in chromosomes 5 and 6 could be responsible for some of the observed biologic differences between *C. parvum* and *C. hominis* [18].

Although the first two genomes of *Cryptosporidium* spp. were sequenced over a decade ago, studies on genome evolution within the *Cryptosporidium* lineage is practically non-existent. As a result, we still have very limited knowledge of the evolution and invasion of *Cryptosporidium* spp. This is largely the result of only a limited number of *Cryptosporidium* species sequenced at the whole genome level. Here, we sequenced the genomes of six *C. ubiquitum* and *C. andersoni* isolates and conducted a comparative genomic analysis of *Cryptosporidium* spp. and other well-studied apicomplexans. We focused on reductive evolution in energy metabolism and differential evolution of invasion-related proteins among *Cryptosporidium* species, as they may be involved in tissue tropism and host specificity.

## Results

### Genome reduction in *Cryptosporidium*

The genomes of three isolates each of *C. ubiquitum* and *C. andersoni* were sequenced. The best assembly was 8.97 Mb in 27 contigs for *C. ubiquitum* and 9.10 Mb in 96 contigs for *C. andersoni* (Additional file 1: Table S1).

Genomes of all *Cryptosporidium* spp. have similar structural features, including genome size, GC content, number of tRNA genes, codon usages, and over-represented DNA sequence motifs in upstream regions of protein-coding genes (Table 1 and Additional file 2: Figure S1). Gene organization of chromosomes is in almost complete synteny and sequences identity is higher between the genomes of *C. ubiquitum* and *C. parvum*. In contrast, the genome sequence of *C. andersoni* is very different from that of *C. parvum* (Additional file 3: Figure S2A). Altogether, 3767 and 3905 protein-coding genes were identified in the *C. ubiquitum* and *C. andersoni* genomes, respectively (Table 1).

**Table 1:** Genomic features of *Cryptosporidium ubiquitum* and *C. andersoni* in comparison with other apicomplexan parasites^a^

	*P. falciparum*	*T. gondii*	*C. parvum*	*C. hominis*	*C. ubiquitum*	*C. andersoni*
Total length (Mb)	22.85	65.67	9.10	8.74	8.97	9.09
No. of super contigs	16	2,263	8	1,422	27	135
GC content (%)	20.0	48.5	30.3	30.9	30.8	28.5
No. of genes	5,542	8,322	3,805	3,886	3,767	3,905
Total length of CDS (Mb)^b^	12.58	20.03	6.83	5.28	6.94	6.86
GC content in CDS (%)	25.0	56.0	31.9	32.7	33.0	30.1
GC content at 3^rd^ position in codons (%)	18.0	59.0	18.0	19.0	20.0	14.0
Mean length of genes (bp)	2,271	2,407	1,720	1,360	1,841	1,757
Gene density (gene/Mb)	242.5	126.7	418.1	444.6	420.0	429.6
Percent coding (%)	55.1	30.5	75.0	60.4	77.4	75.5
No. of genes with intron	3,055	6,729	163	8	758	832
Genes with intron (%)	55.1	80.9	4.2	0.02^c^	20.1	21.3
No. of tRNA	72	174	45	46	45	44
No. of tRNA^met^	2	8	2	2	2	2
Proteins with signal peptide	638	759	397	421	399	309
Proteins with transmembrane domain	1,754	1,103	832	769	772	839
Proteins with GPI anchor	62	255	63	51	50	47

^a^Source of data: *Plasmodium falciparum*: PlasmoDB release-11.1; *Toxoplasma gondii*: ToxoDB release-11.0; *C. parvum* and *C. hominis*: CryptoDB release-6.0

^b^Coding regions excluding intron sequences

^c^Estimated to be 5–20% by Xu et al., [4]

**Fig. 1:**
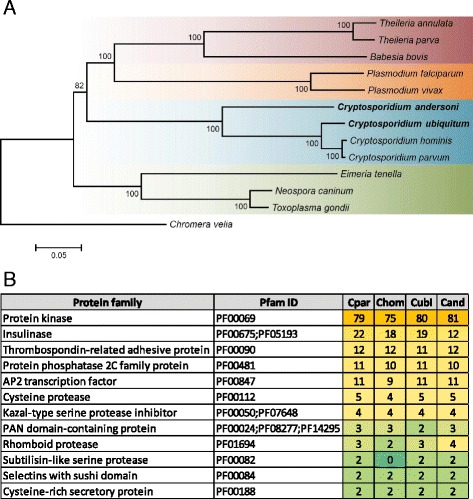
Genomic features and phylogenetic relationship of *Cryptosporidium* spp. **a** Phylogenetic relationship between *Cryptosporidium* spp. and other common apicomplexan parasites based on neighbor-joining analysis of sequences of 100 shared proteins. A concatenated sequences from the free-living photosynthetic chromerid, *Chromera velia,* was used to root the tree. **b** Comparison of major invasion-related protein families among *Cryptosporidium* species. The number of each protein family was identified based on Pfam domain search only, which may differs from the result of ortholog comparisons. Abbreviation of *Cryptosporidium* spp.: Cpar: *Cryptosporidium parvum*; Chom: *C. hominis*; Cubi: *C. ubiquitum*; Cand: *C. andersoni*

The *Cryptosporidium* species under analysis share a large group of orthologs, with only a small number of species-specific genes. As expected, the number of species-specific genes in *C. andersoni* is apparently bigger than that among intestinal species (Additional file 3: Figure S2B). The divergent nature of *C. andersoni* is supported by phylogenetic analysis of 100 orthologs from *Cryptosporidium* spp. and other common apicomplexan parasites (Fig. 1a). All *Cryptosporidium* species have similar numbers and components of major protein families, except for *C. andersoni*, which has fewer genes encoding insulinase-like peptidases (Fig. 1b).

The metabolic capabilities of the four *Cryptosporidium* spp. are dramatically reduced compared with *P. falciparum* and *T. gondii*. This is especially reflected in carbohydrate, amino acid, and energy metabolism, with a limited *de novo* biosynthesis and overall reliance on the hosts for basic nutrients by *Cryptosporidium* spp. (Table 2). Other reductive evolutionary features in *Cryptosporidium* spp. include reduced gene numbers and length and increased gene density (Table 1), which were previously observed in *C. parvum* and *C. hominis* genomes [4, 5]. As expected, no apicoplast and mitochondrial genomes were detected in any *Cryptosporidium* species.

**Table 2:** Comparison of essential metabolic pathways among *Cryptosporidium* spp. and other common apicomplexan parasites^a^

Category	Metabolic pathway	Cpar	Chom	Cubi	Cand	Pfal	Tgon
Carbohydrate and energy metabolism	Glycolysis	+	+	+	+	+	+
	Methylcitrate cycle	-	-	-	-	-	+
	TCA cycle	-	-	-	+	+	+
	Pentose phosphate pathway	-	-	-	-	+	+
	Shikimate biosynthesis	-	-	-	-	+	+
	Folate biosynthesis	-	-	-	-	+	+
	Synthesis of tetrahydrobiopterin/dihydrobiopterin/molybdopterin	-	-	-	-	-	+
	Galactose metabolism	-	-	-	-	-	+
	Synthesis of starch	+	+	+	+	-	+
	Synthesis of trehalose	+	+	+	+	-	+
	Synthesis of 1,3-beta-glucan	-	-	-	-	-	+
	UDP-Glc < - > UDP-Gal	+	+	+	+	-	+
	GDP-Man < - > GDP-Fuc	-	-	-	-	+	+
	UDP-Glc - > UDP-GlcA - > UDP-Xyl	+	+	+	+	-	-
	Synthesis of mannitol from fructose	+	+	+	+	-	-
	Fatty acid biosynthesis in cytosol (FAS I)	+	+	+	+	-	+
	Fatty acid biosynthesis in apicoplast (FAS II)	-	-	-	-	+	+
	Fatty acid degradation	-	-	-	-	-	+
	Oxidative phosphorylation (NADH dehydrogenase)	+	+	+	+	+	+
	Oxidative phosphorylation (Complex II)	-	-	-	+	+	+
	Oxidative phosphorylation (Complex III)	-	-	-	1 subunit	+	+
	Oxidative phosphorylation (Complex IV)	-	-	-	-	+	+
	F-ATPase	2 subunit	2 subunit	2 subunit	+	+	+
	Alternative oxidase (AOX)	+	+	+	+	-	-
	Glyoxalase metabolism producing D-lactate	-	-	-	-	+	+
	Synthesis of isoprene (MEP/DOXP)	-	-	-	-	+	+
	Synthesis of farnesyl/polyprenyl diphosphate	+	+	-	+	+	+
Nucleotide metabolism	Synthesis of purine rings de novo	-	-	-	-	-	-
	IMP - > XMP - > GMP	+	+	+	-	+	+
	Synthesis of pyrimidine de novo	-	-	-	-	+	+
Amino acid metabolism	Synthesis of alanine from pyruvate	-	-	-	-	-	+
	Synthesis of glutamate from nitrite/nitrate	-	-	-	-	+	+
	Conversion from glutamate to glutamine	+	+	+	+	+	+
	Synthesis of aspartate from oxaloacetate and glutamate	-	-	-	-	+	+
	Conversion from aspartate to asparagine	+	+	+	-	+	+
	Conversion from glutamate to proline	+	+	+	+	-	+
	Synthesis of serine from glycerate/glycerol phosphate	-	-	-	-	-	+
	Conversion from serine to cysteine	-	-	-	-	-	+
	Conversion from serine to glycine	+	+	+	+	+	+
	Recycle homocysteine into methionine	-	-	-	-	+	+
	Synthesis of lysine from aspartate	-	-	-	-	-	+
	Synthesis of threonine from aspartate	-	-	-	-	-	+
	Synthesis of ornithine from arginine	-	-	-	-	+	-
	Synthesis of ornithine from proline	-	-	-	-	+	+
	Synthesis of polyamine from ornithine	-	-	-	-	+	-
	Polyamine pathway backward	+	+	+	+	-	+
	Degradation of branch-chain amino acids	-	-	-	-	-	+
	Synthesis of tryptophan	+	+	+	-	-	-
	Aromatic amino acid hydroxylases (AAAH)	-	-	-	-	-	+
Vitamin and others	Synthesis of ubiquinone (Coenzyme Q)	+	+	-	+	+	+
	Synthesis of Fe-S cluster	+	+	+	+	+	+
	Synthesis of heme	-	-	-	-	+	+
	Synthesis of thiamine (Vitamin B1)	-	-	-	-	+	-
	Conversion from thiamine to thiamine pyrophosphate (TPP)	-	-	-	-	+	+
	Synthesis of FMN/FAD from riboflavin	-	-	-	-	+	+
	Synthesis of pyridoxal phosphate (Vitamin B6) de novo	-	-	-	-	+	+
	Synthesis of NAD(P) + de novo from nicotinate/nicotinamide	-	-	-	-	+	+
	Synthesis of pantothenate from valine	-	-	-	-	-	+
	Synthesis of CoA from pantothenate	+	+	+	+	+	+
	Synthesis of lipoic acid de novo in apicoplast	-	-	-	-	+	+
	Salvage of lipoic acid in mitochondria	-	-	-	+	+	+
	Synthesis of porphyrin/cytochrome proteins	-	-	-	-	+	+

^a^Plus symbol denotes that the essential enzymes for the pathway were identified, whereas minus symbol denotes that the essential enzymes for pathways were absent

Cpar *Cryptosporidium parvum*, Chom *C. hominis*, Cubi *C. ubiquitum*, Cand *C. andersoni*, Pfal *Plasmodium falciparum* Pfal, Tgon *Toxoplasma gondii*

### Divergent mitosome metabolism among *Cryptosporidium* spp.

**Fig. 2:**
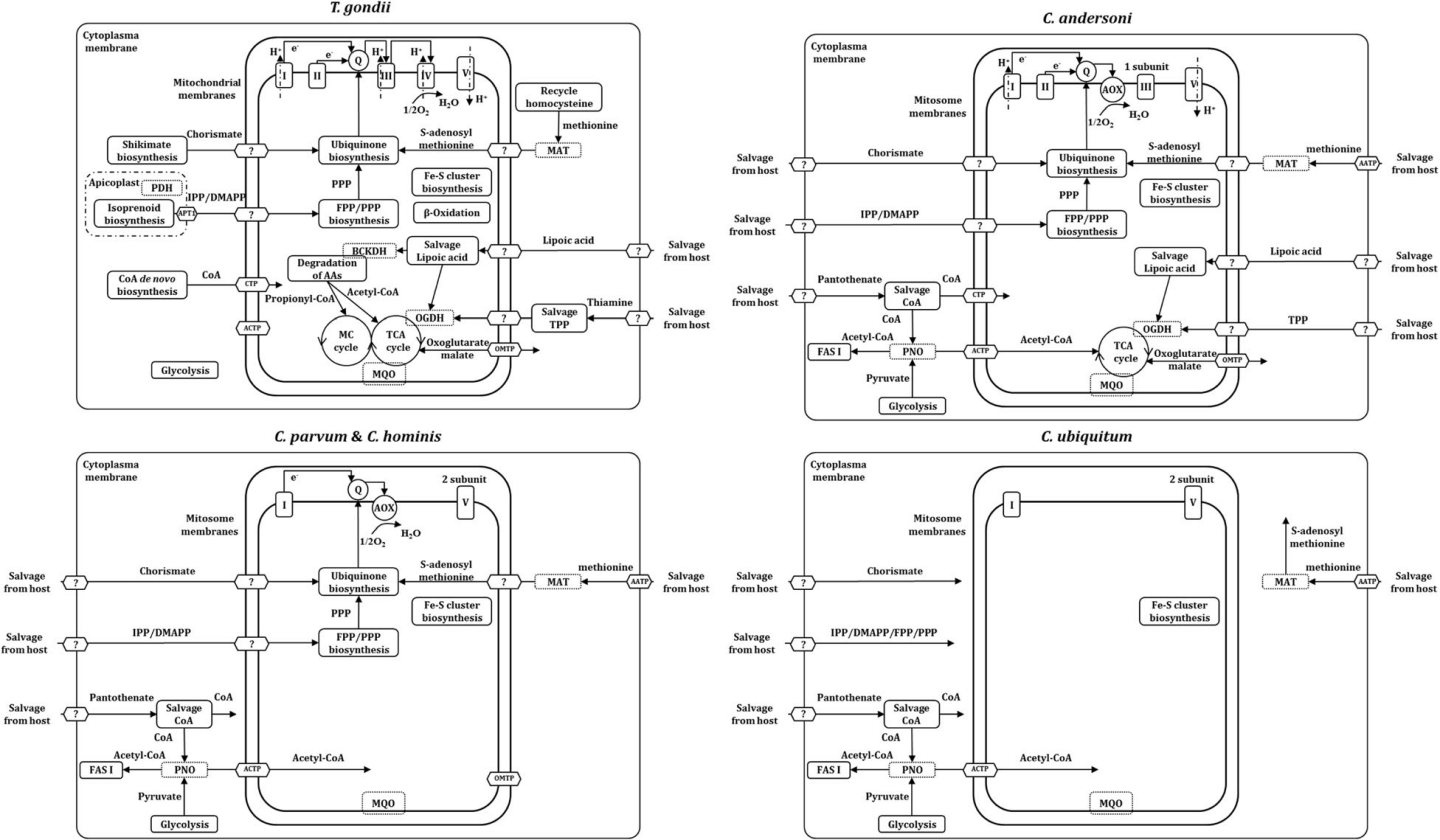
Reductive evolution in mitosome metabolism among *Cryptosporidium* spp. in comparison with *Toxoplasma gondii*. Abbreviation of enzymes: AOX: alternative oxidase; PDH: pyruvate dehydrogenase complex; PNO: pyruvate:NADP(+) oxidoreductase; MAT: methionine adenosyl transferase; OGDH: oxoglutarate dehydrogenase complex; MQO: malate:quinone oxidoreductase; BCKDH: branched-chain ketoacid dehydrogenase complex. Abbreviation of metabolites: Q: ubiquinone (coenzyme Q); CoA: coenzyme A; IPP: isopentenyl diphosphate; DMAPP: dimethylallyl diphosphate; FPP: farnesyl diphosphate; PPP: polyprenyl diphosphate; MC: 2-methylcitrate; TCA: tricarboxylic acid; TPP: thiamine pyrophosphate. Abbreviation of transporter proteins: APT1: apicoplast phosphate translocator; CTP: CoA transporter protein; ACTP: acetyl-CoA transporter protein; OMTP: oxoglutarate/malate transporter protein

Compared with the canonical aerobic mitochondrion in most eukaryotic organisms, the mitosome of *Cryptosporidium* spp. has undergone remarkable reduction in size and function probably because of the anaerobic environment in the digestive tract [19]. The comparative analysis of metabolism of *C. ubiquitum, C. andersoni* and other *Cryptosporidium* spp. shows that mitosome metabolism, including the electron transport chain, has gone through progressive reductions within the *Cryptosporidium* genus (Fig. 2).

#### TCA cycle and related metabolism in *C. andersoni*

Unlike intestinal *Cryptosporidium* spp., *C. andersoni* possesses a complete set of enzymes involved in the TCA cycle and related metabolism, such as the substrate transport system and salvage of the cofactor lipoic acid (Table 2, Fig. 2). In *Plasmodium falciparum* and *Toxoplasma gondii*, pyruvate derived from glycolysis is oxidated into acetyl-CoA by a multi-protein enzyme in the apicoplast, pyruvate dehydrogenase complex (PDH) [6]. *Cryptosporidium* spp. do not have this complex, but possess an alternative pyruvate:NADP+ oxidoreductase (PNO) localized in the cytosol [20]. The product, acetyl-CoA presumably is delivered into mitosomes through the transporter protein embedded on the mitosome membrane in *C. andersoni*. In other *Cryptosporidium* spp., acetyl-CoA is primarily involved in metabolic pathways in the cytosol, such as type I fatty acid biosynthesis, although this pathway was also detected in *C. andersoni*. The oxoglutarate dehydrogenase complex (OGDH) from the TCA cycle can become active when it couples with two cofactors, thiamine pyrophosphate (TPP) and lipoic acid. Among apicomplexans, only *P. falciparum* has the ability to synthesize thiamine *de novo* [21]. *C. andersoni* has to salvage TPP directly from the host, although its transporter has not been identified. In addition to synthesizing lipoic acid *de novo* in the apicoplast, *P. falciparum* and *T. gondii* can salvage lipoic acid from the host into the mitochondrion and conjugate it onto OGDH through the lipoate-protein ligase [22]. The gene encoding this ligase is present in the *C. andersoni* genome, indicating that *C. andersoni* salvages host lipoic acid and utilizes it presumably in mitosomes. Unlike *T. gondii,* which possesses *de novo* biosynthesis of CoA, all *Cryptosporidium* spp. salvage pantothenate from host cells and convert it into CoA.

#### Absence of polyisoprenoid biosynthesis in *C. ubiquitum*

In *T. gondii*, two 5-carbon isoprene units, isopentenyl diphosphate (IPP) and dimethylallyl diphosphate (DMAPP), are synthesized *de novo* in the apicoplast from glycolysis-derived phosphonel pyruvate and dihydroxyacetone phosphate through the chloroplast-type MEP/DOXP pathway [6]. Both IPP and DMAPP are transported into the mitochondrion and condensed into farnesyl diphosphate (FPP) and polyprenyl diphosphate (PPP) [21, 23]. All *Cryptosporidium* spp. lack not only the apicoplast but also the genes coding enzymes for *de novo* isoprenoid biosynthesis (Table 2, Fig. 2). Within the subsequent mitosome polyisoprenoid anabolic pathway, two essential enzymes, FPP synthase and polyprenyl synthase, are absent in the predicted proteome of *C. ubiquitum* but present in other *Cryptosporidium* spp. The genes (cgd4_2550, cgd7_3730) encoding these two enzymes were shown transcribed in *C. parvum* in vitro, especially during early (2–6 h) infection [24]. Artz et al. suggested that the enzyme encoded by cgd4_2550 is a non-specific polyprenyl pyrophosphate synthase rather than FPP synthase only [25]. These two enzymes may cooperatively synthesize pyrophosphoric polyisoprenoids in *C. parvum*. As PPP is an essential substrate for the biosynthesis of ubiquinone, the absence of polyisprenoid biosynthesis in *C. ubiquitum* is consistent with its loss of ubiquinone biosynthesis (see below). In addition to participating in the biosynthesis of ubiquinone, isoprenoids such as FPP and PPP are involved in signaling pathways, post-translational modifications of proteins, and isoprenylation of tRNAs [6]. *C. ubiquitum* possesses enzymes involved in the isoprenylation of both proteins and tRNAs, suggesting that it may salvage FPP and PPP in addition to IPP and DMAPP from the host.

The absence in *C. ubiquitum* of three mitosome carrier proteins possessed by *C. parvum* further demonstrates that mitosome metabolic capabilities in *C. ubiquitum* are more limited than those of other *Cryptosporidium* spp. (Table 3). Because of the existence of more metabolic pathways within mitosomes, *C. andersoni* has several more mitosome transporter proteins than other *Cryptosporidium* spp. In addition, *C. andersoni* possesses two more amino acid transporters and one more potassium transporter than other *Cryptosporidium* spp. (Table 3).

**Table 3:** Putative transporters in *Cryptosporidium* spp. and other common apicomplexan parasites^a^

Substrates	Cellular location	Cpar	Chom	Cubi	Cand	Pfal	Tgon
Hexose		2	2	2	2	2	5
Triose phosphate	Plasma/Apicoplast membrane	8	5	8	8	4	4
Amino acids	Plasma membrane	10	10	10	12	1	6
Nucleobase/nucleoside	Plasma membrane	1	1	1	1	4	4
Nucleotide-sugar	Plasma membrane	3	3	3	2	1	4
Folate/pterine	Plasma membrane	1	1	1	1	2	7
Formate/nitrite		0	0	0	0	1	3
GABA (aminobutanoate)	Plasma/Mitochondrial membrane	0	0	0	0	2	5
Acetyl-CoA		1	0	1	1	1	1
Chloride		0	0	0	0	0	2
Inorganic phosphate		0	0	0	0	1	1
Sulfate		1	1	1	1	1	4
Sodium/potassium/calcium		2	2	2	3	0	9
Zinc		2	2	2	2	2	4
Copper		1	1	1	1	2	3
Choline	Plasma membrane	0	0	0	0	1	2
Cadmium/zinc/cobalt (efflux)	Plasma membrane	1	1	1	1	1	1
Glycerol/water	Plasma membrane	0	0	0	0	2	2
ABC transporter^b^	Plasma membrane	21	18	21	21	16	24
Mitochondrial carrier^b^	Mitochondrial membrane	9	7	6	13	14	21

^a^The detection of these transporter proteins was based on the Pfam search results

^b^ABC transporters and mitochondrial carriers have a broad range of substrates

Cpar *Cryptosporidium parvum*, Chom *C. hominis*, Cubi *C. ubiquitum*, Cand *C. andersoni*, Pfal *Plasmodium falciparum* Pfal, Tgon *Toxoplasma gondii*

#### Progressive reduction in electron transport chain in *Cryptosporidium* spp.

Within the oxidative phosphorylation pathway on the inner mitochondrial membrane, the classical NADH dehydrogenase multi-protein complex, named complex I, is substituted by an alternative single NADH dehydrogenase in most apicomplexan parasites [21]. Similar to *P. falciparum* and *T. gondii*, which use three other multi-protein complexes (II-IV) to transfer electrons to oxygen, *C. andersoni* has the complete complex II, succinate dehydrogenase, which is also involved in the TCA cycle, and one subunit of complex III, ubiquinol-cytochrome *c* oxidoreductase. In contrast, *C. parvum*, *C. hominis*, and *C. ubiquitum* have lost these enzyme complexes entirely (Table 2, Fig. 2). With electrons transported through the chain, a proton gradient is generated across the inner mitochondrial membrane and the energy generated can be used to produce ATP by the ATP synthase (also known as complex V). Similar to *P. falciparum* and *T. gondii*, *C. andersoni* possesses a complete F-type ATP synthase, but only two subunits (subunit α and β) of this enzyme are present in *C. parvum*, *C. hominis* and *C. ubiquitum*.

Ubiquinone, also known as coenzyme Q, is responsible for transferring electrons from complex I or complex II to complex III. It is synthesized from chorismate, which has to be salvaged from the host because of the absence of the shikimate pathway in *Cryptosporidium* spp., together with two other substrates polyisoprenoid and adenosyl methionine [21]. *C. parvum*, *C. hominis*, and *C. andersoni* possess all enzymes and proteins involved in the ubiquinone biosynthesis [21]. Most of these enzymes have experimental expression evidence in *C. parvum* and show a higher expression level at the end (36–72 h) of the in vitro infection [24]. The absence of five additional enzymes suggests that *C. ubiquitum* lacks the capability of ubiquinone biosynthesis (Table 2, Fig. 2).

In addition to the conventional mitochondrial electron transport system, there is also a cytochrome-independent system executed by a cyanide-insensitive alternative oxidase (AOX), passing electrons from ubiquinone directly to oxygen to form water in higher plants, fungi and several protozoa [26–28]. All *Cryptosporidium* spp. possess orthologs of AOX [4, 5], with the exception of *C. ubiquitum*. This alternative pathway does not couple the proton transport across the inner mitosome membrane, indicating that energy production is reduced in intestinal *Cryptosporidium* spp. AOX in *C. parvum* can be inhibited by salicylhydroxamic acid and 8-hydroxyquinoline, affecting the growth of the parasite [27]. The mammalian bloodstream form of *Trypanosoma brucei* depends entirely on the AOX pathway for electron transport, making AOX an attractive target for drug development [29, 30]. The absence of AOX in *C. ubiquitum* suggests that the electron transport chain is totally lost in this apicomplexan parasite (Table 2, Fig. 2).

Unexpectedly, the gene encoding malate:quinone oxidoreductase (MQO), the enzyme involved in both the TCA cycle and electron transport chain, is present in most apicomplexans including *C. parvum*, *C. hominis* and *C. ubiquitum* (Fig. 2). MQO was presumably gained by the ancestors of apicomplexans, chromerids, and dinoflagellates from bacteria though lateral gene transfer [8].

### Reductive evolution in biosynthesis of *N*-glycan and GPI-anchor precursors in *Cryptosporidium* spp.

**Fig. 3:**
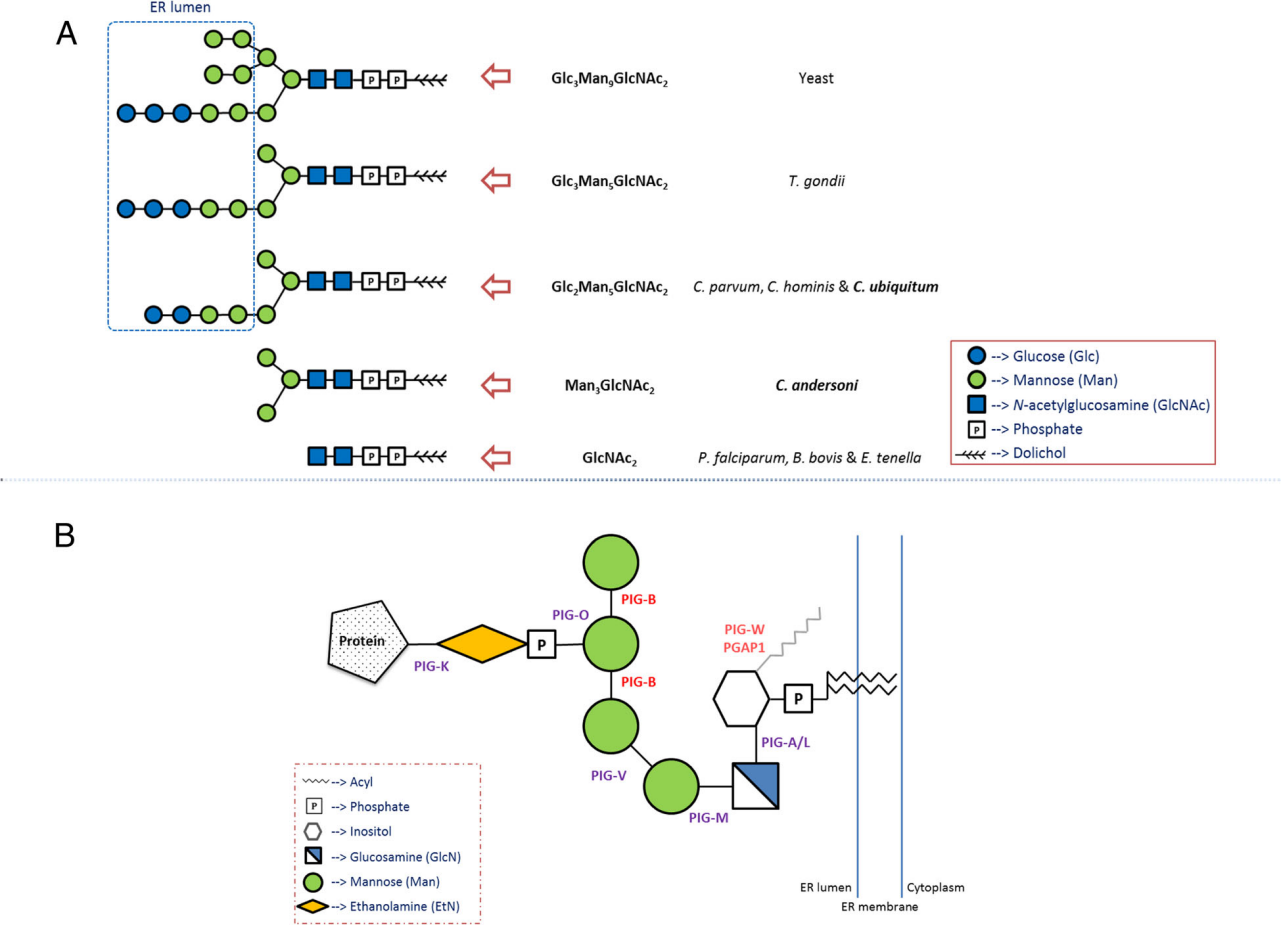
Diversity in post-translational modifications of proteins among *Cryptosporidium* spp. A) Divergent N-glycan precursors in different *Cryptosporidium* spp. and other apicomplexan parasites. B) Core structure of GPI-anchor and the critical enzymes involved in its biosynthesis. PIG-B is absent in *Cryptosporidium ubiquitum* but present in other *Cryptosporidium* spp., whereas PIG-W and PGAP1 are absent in all *Cryptosporidium* spp.

Asparagine (*N*)-linked glycosylation is a common post-translational modification of proteins and the biosynthesis of *N*-glycans has been shown to be different among apicomplexan parasites [31]. Due to the secondary loss of *Alg* genes, apicomplexans differ from each other in the length of *N*-glycan precursors; the length of oligosaccharide chain of *N*-glycan precursors in *Cryptosporidium* spp. is shorter than that in *T. gondii* but longer than in *P. falciparum* (Fig. 3a). Among *Cryptosporidium* spp., intestinal species *C. parvum*, *C. hominis* and *C. ubiquitum* possess nine sugars in *N*-glycan precursors, whereas the gastric species *C. andersoni* has only five sugars. The addition of two mannose molecules and two glucose molecules onto the core structure of *N*-glycan precursor is lost in *C. andersoni*.

A large number of surface proteins attach to the cell membrane via the glycosylphosphatidylinositol (GPI) anchor [32]. The construction of GPI-anchors occurs in three stages: i) biosynthesis of a GPI precursor in the endoplasmic reticulum (ER) membrane, ii) attachment of the GPI to the C-terminus of a newly synthesized protein in the ER lumen, and iii) lipid remodeling and/or carbohydrate side-chain modifications in ER and *Golgi* lumens. The critical mannosyltransferase (PIG-B) catalyzing the addition of the third and fourth mannose onto the GPI-anchor precursor located in the ER lumen is present in *C. parvum*, *C. hominis* and *C. andersoni* but absent in *C. ubiquitum* (Fig. 3b). The transcription of the PIG-B encoding gene (cgd3_3590) has been demonstrated in *C. parvum*, with a high level of expression at the end of in vitro infection [24]. In addition, unlike in other apicomplexan parasites, the acylation (mediated by PIG-W) and de-acylation (mediated by PGAP1) of inositol during the construction of GPI-anchor precursors in the ER lumen are absent in all *Cryptosporidium* spp.

### Reductive evolutions in other metabolic pathways

The gene encoding tryptophan synthase (cgd5_4560) catalyzing biosynthesis of tryptophan from serine and indole was identified in intestinal *Cryptosporidium* spp. but not in the gastric species *C. andersoni*. It has been suggested that intestinal bacteria can provide indole to *C. parvum* and *C. hominis* whereas this bacterial community does not exist in the stomach of the host, leading to the absence of tryptophan biosynthesis in gastric *Cryptosporidium* spp. [21]. Similarly, the gene encoding asparagine synthase A (AsnA) (cgd5_4540), which converts aspartate into asparagine, is only absent in *C. andersoni*.

All apicomplexan parasites are unable to synthesize purine rings *de novo* and have to salvage them from the host. Only one purine nucleotide transporter was detected in *Cryptosporidium* spp. and it probably transports adenosine only [21]. *C. parvum*, *C. hominis* and *C. ubiquitum* convert AMP into GMP through IMP dehydrogenase (cgd6_20) and GMP synthase (cgd5_4520), which have not been identified in *C. andersoni*.

### Differential evolution in invasion-related proteins

**Fig. 4:**
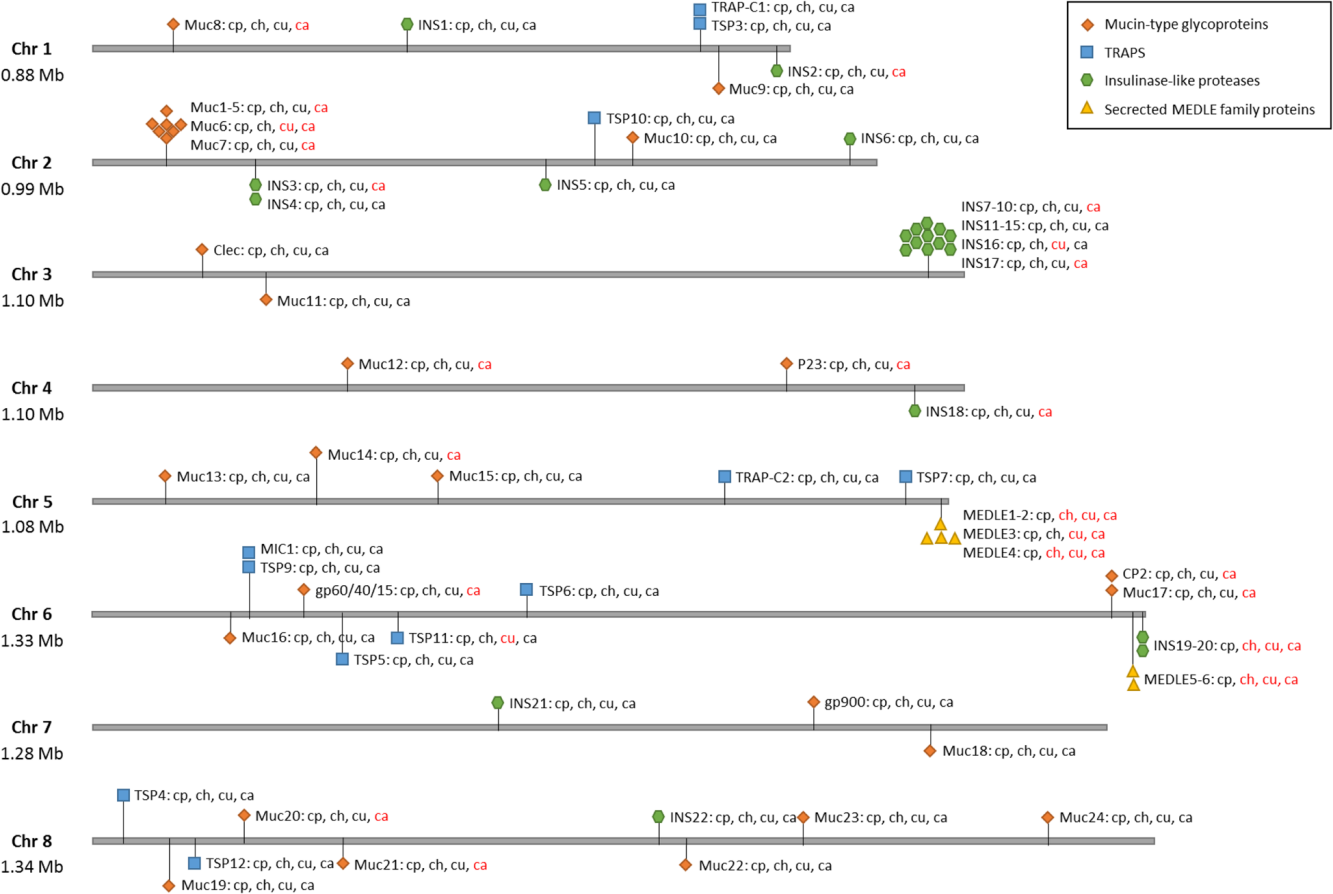
Diversity in invasion-related secreted protein and peptidase families among *Cryptosporidium* species. Abbreviation of *Cryptosporidium* spp.: cp: *Cryptosporidium parvum*; ch: *C. hominis*; cu: *C. ubiquitum*; ca: *C. andersoni*. The species name in red represents the absence of this family member

Apicomplexan parasites possess several unique secretory organelles such as the rhoptry, microneme, and dense granules, which secret various catalytic proteins for host cell adhesion and invasion [33]. Among secreted proteins of *Cryptosporidium* spp., two protein families, mucin-type glycoproteins and thrombospondin-related adhesive proteins (TRAPs), are considered essential to host cell attachment [34, 35]. The genes encoding mucin-type glycoproteins associated with adhesion, such as gp900, gp60/40/15, P23, P30, CP2, and Clec, were compared among the four *Cryptosporidium* species and results of the comparison have shown a high divergence of *C. andersoni* from other species in compositions of these genes (Fig. 4 and Additional file 4: Table S2). In *C. parvum*, the gp60/40/15 complex, which is absent in *C. andersoni*, is probably translated from one single mRNA, glycosylated, and proteolytically processed into two smaller glycoproteins. The latter are localized at the surface or apical region of the parasite and thought to be involved in attachment and invasion [34]. P23, a surface protein that induces antibody responses in animal hosts, is absent in *C. andersoni* but present in other *Cryptosporidium* spp. [34]. CP2, the membrane associated protein that is also absent in *C. andersoni*, has been shown to be localized in the parasitophorous vacuole membrane (PVM) and is probably involved in host cell invasion or PVM integrity [36]. In addition, seven small mucin genes (*muc*1-7) located in tandem in chromosome 2 of *C. parvum* are expressed during intracellular development and products of at least two of them, Muc4 and Muc5, are associated with host cell attachment and invasion [37]. Among them, Muc6 is absent in *C. ubiquitum* and none of them are present in *C. andersoni*. A major characteristic of these mucin proteins is their low similarity in amino acid sequences. Among the species-specific proteins, 5 and 30 are O-linked mucin-type glycoproteins in *C. ubiquitum* and *C. andersoni*, respectively (Additional file 5: Table S3). Some of them are probably responsible for host specificity in *Cryptosporidium* spp. In addition to host cell attachment, mucin-type glycoproteins may contribute to the tethering of sporozoites to the inner surface of the oocyst walls [38].

TRAPs, the best-characterized microneme proteins for gliding motility and host cell invasion in apicomplexans, usually contain two types of adhesive domains: the von Willebrand factor type A (VWA) domain and thrombospondin 1 (TSP1) domain, such as the TRAP of *P. falciparum* and MIC2 of *T. gondii* [39]. A screening of the TSP1 domain revealed that *C. parvum* possesses 12 paralogous genes (CpTSP1-12), which all have putative signal peptides and one or more TSP1 domains [40]. There are no VWA domains but other modules such as Kringle and epidermal growth factor (EGF) were detected within these TRAP genes. CpTSP8, previously known as CpMIC1 containing three TSP1 repeats and one EGF domain, is translocated to the surface of the parasite together with other microneme proteins [41]. All of these TRAP genes are present in the *C. ubiquitum* and *C. andersoni* genomes with the exception of TSP11, which is located in chromosome 6 and highly expressed in both early and late stages of *C. parvum* infection [40], is absent in *C. ubiquitum* (Fig. 4 and Additional file 4: Table S2).

A variety of secreted proteases and protein kinases in secretory organelles of apicomplexans are involved in processing invasion-related proteins or modifying host cell activities, enabling the evasion of host immune system during invasion [42]. Two members of the insulinase-like proteases containing the peptidase family M16 domain located in the subtelomeric region of chromosome 6 of *C. parvum* have been shown to be lost in the *C. hominis* genome. This is one of the few differences in gene content between these two closely related species with different host specificity [18]. In addition to the absences of these two subtelomeric genes on chromosome 6, one member (the ortholog of cgd3_4270, which has 90% nucleotide sequence similarity to cgd3_4260) of another insulinase gene cluster in the subtelomeric region of chromosome 3 of *C. parvum* is absent in *C. ubiquitum*. Additional paucity of this gene family was seen in *C. andersoni,* especially in genes located in subtelomeric regions. No new insulinase-like genes were identified in *C. ubiquitum,* although *C. andersoni* appears to have two or three new ones (Fig. 4 and Additional file 4: Table S2).

Another major genetic difference between genomes of *C. parvum* and *C. hominis* is in the number of genes encoding the MEDLE family surface proteins in the 3′ subtelomeric regions of chromosomes 5 and 6. Five of the six MEDLE family protein genes in *C. parvum* have no orthologs in *C. hominis* [18]. The two subtelomeric regions are both absent in genomes of *C. ubiquitum* and *C. andersoni* (Fig. 4 and Additional file 4: Table S2). Although the specific functions of these proteins have not been determined, most of them have a signal peptide.

Rhomboids, a family of intra-membrane serine proteases, are ubiquitously present in apicomplexan parasites and responsible for the cleavage of secreted adhesive proteins [43]. One additional rhomboid gene was detected in the genome of *C. andersoni* compared with other *Cryptosporidium* spp. (Additional file 4: Table S2).

## Discussion

**Fig. 5:**
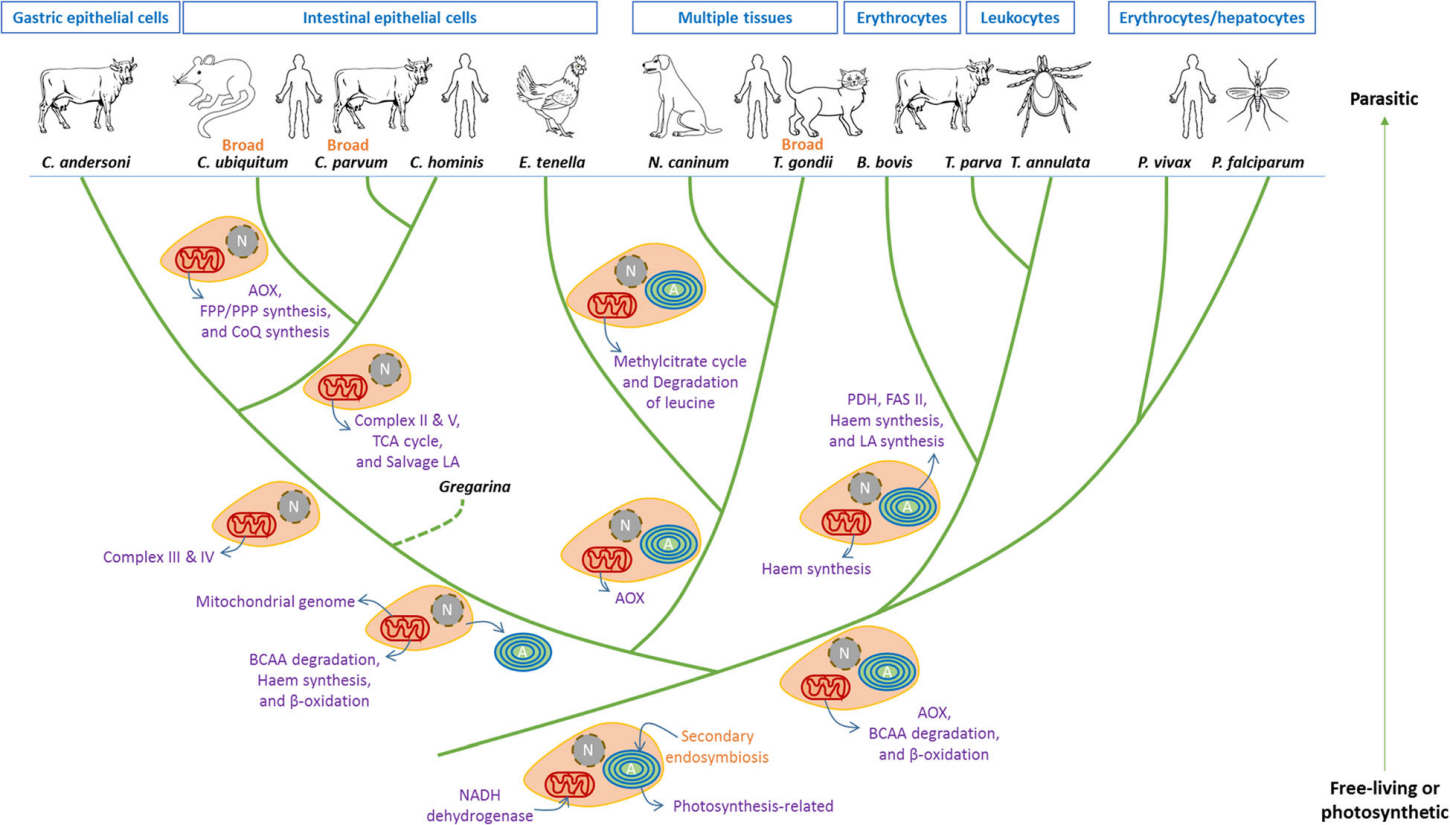
Reductive evolution of organelle-derived metabolism among major apicomplexan lineages. Abbreviation: AOX: alternative oxidase; FPP: farnesyl diphosphate; PPP: polyprenyl diphosphate; CoQ: coenzyme Q; TCA: tricarboxylic acid; LA: lipoic acid; BCAA: branched-chain amino acid; PDH: pyruvate dehydrogenase complex; FAS: fatty acid synthase

Whole genome sequencing and comparative genomic analysis have revealed a progressive reduction of mitosome metabolism in the genus *Cryptosporidium*, including major energy production mechanism, the electron transport chain, and the associated ancillary pathways. As shown in this study, like the genetically related *C. muris* [8], *C. andersoni* uses both the TCA cycle and glycolysis for energy metabolism and has a near conventional oxidative phosphorylation system. In contrast, *C. parvum* and *C. hominis* do not have the TCA cycle and possess an alternative oxidative phosphorylation chain, whereas *C. ubiquitum* has further lost the entire electron transport chain and the associated biosynthesis of ubiquinone and polyisoprenoids. It has been shown that *Cryptosporidium* spp. and gregarines differ from other apicomplexans in energy metabolism because of the absence of the intracellular organelle apicoplast and the presence of an alternative electron transport system. Data from this study now show a progressive reductive evolution in energy metabolism within the *Cryptosporidium*/gregarine lineage of apicomplexans. Thus far, energy metabolism and electron transport chain appear remarkably conserved in other lineages of apicomplexans (Fig. 5).

It is generally accepted that the ancestor of apicomplexan parasites obtained the mitochondrion and apicoplast through primary and secondary endosymbiosis, respectively [22]. During the reductive evolution process, most of the organelle metabolism-related genes have been progressively lost or horizontally transferred to nuclear genomes as different lineages adjust to new life strategies that differ from the photosynthetic or free-living lifestyle [2, 7]. *Cryptosporidium* spp. represent the extreme of apicomplexans, with the total loss of the apicoplast and having only a mitochondrial replica without the associated genome. The progressive losses of energy generation pathways and electron transport chains in *Cryptosporidium* spp. are probably results of adaptation to different niches of parasitism. As gregarines have retained several common features, such as the presence of a TCA cycle and a conventional electron transport chain, they probably represent ancestry members of the *Cryptosporidium*/gregarine lineage. Within the *Cryptosporidium* genus, *C. andersoni* appears to be an earlier member of the genus *Cryptosporidium*, whereas *C. ubiquitum* is probably a recent one (Fig. 5). Studies with molecular clocks are needed to confirm this theory.

The loss of enzymes involved in the biosynthesis of ubiquinone and polyisoprenoids in *C. ubiquitum* supports the conclusion on progressive evolution of energy metabolism and electron transport systems in the *Cryptosporidium*/gregarine lineage. Accompanying the loss of the conventional and alternative electron transport systems, *C. ubiquitum* has lost the ability to synthesize polyisoprenoids, which are substrates in ubiquinone biosynthesis, and ubiquinone itself, which is part of the conventional electron transport chain. By inspection of Mauve genome alignments, most of the genes that are absent in *C. ubiquitum* but present in *C. parvum* and *C. hominis* have syntenic nucleotide sequences in the *C. ubiquitum* genome, suggesting the occurrence of secondary losses of these functional genes in *C. ubiquitum* during species evolution. In agreement with this, *C. andersoni*, which has a TCA cycle, possesses several transporters for TCA substrates (such as acetyl CoA, oxoglutarate malate, and OGDH), more mitosome carrier proteins, and a salvage system for the cofactor lipoic acid. *C. ubiquitum* probably needs to salvage isoprenoids from the host for tRNA isoprenylation via enzyme miaA, which together with its downstream enzyme, miaB, is present in all *Cryptosporidium* genomes. Because isoprenylation of tRNA in apicomplexans occur mostly in the apicoplast [6], the ancestor of *Cryptosporidium* spp. probably had an apicoplast.

Reductive evolution apparently occurs in other metabolism processes in *Cryptosporidium* spp. Highly divergent *N*-glycan precursors have been seen among protists due to the secondary loss of *Alg* genes during reductive evolution [31]. Within the *Cryptosporidium* genus, the *N*-glycan precursor of the gastric *C. andersoni* has four fewer oligosaccharides than intestinal *Cryptosporidium* spp. This is the first time divergent *N*-glycan precursors are seen within a single genus of Apicomplexa, suggesting that the *Alg* genes are progressively lost after the emergence of *Cryptosporidium* spp. Similarly, the enzyme involved in the addition of the third mannose to the GPI anchor core structure, mannosyltransferase, is absent from the *C. ubiquitum* genome. Previously, mannosyltransferase was shown to be missing in *Giardia* and *Entamoeba*, while all enzymes in this pathway are absent in *Trichomonas* [31]. Recently, an absence of mannosyltransferases was detected in *Eimeria falciformis* [44].

*Cryptosporidium* spp. differ from each other in host specificity and tissue tropism. Presumably, the attachment and invasion of host cells are determinants for host specificity and tissue tropism, although differences in mitosome metabolism are probably also involved in the latter. Due to the relatively simple oral-fecal life cycle and the epicellular position of parasitism in restricted host cell type, the repertoire of invasion-related proteins is significantly reduced in *Cryptosporidium* spp. compared with other apicomplexans [45]. Thus far, the most obvious genetic differences between *C. parvum* and *C. hominis* are in the number of paralogous genes encoding secreted MEDLE family proteins and insulinase-like proteases in 3’subtelomeric regions of chromosomes 5 and 6 [18]. The role of these proteins in host specificity of *Cryptosporidium* spp. is supported by observations in this study, as *C. ubiquitum* and *C. andersoni* have lost altogether regions encoding these proteins. *C. andersoni* has further lost many genes of the insulinase-like proteases elsewhere in its genome. Mucin-type glycoproteins probably also play an important role in host specificity and tissue tropism, as they have shown high diversity in gene composition and sequences among *Cryptosporidium* spp., especially between the intestinal and gastric *Cryptosporidium* species. Other surface proteins such as TRAPs are known to be involved in invasion, but their role in host specificity determination is probably limited, as all *Cryptosporidium* spp. largely have the same class and number of TRAPs.

Results of this comparative genomic analysis have revealed drastic genetic differences between gastric and intestinal *Cryptosporidium* species. For example, *C. andersoni* differs from *C. parvum*, *C. hominis*, and *C. ubiquitum* significantly in not only genome organizations but also mitosome metabolism and invasion-associated secreted proteins and peptidases. Previously, gastric and intestinal *Cryptosporidium* species formed separate clades in phylogenetic analysis of the small subunit rRNA gene [46]. In addition to taxonomic implications, these findings suggest that the two groups of pathogens may require different strategies in the development of therapeutic agents. For example, *Cryptosporidium* spp. were known to use mainly glycolysis for energy metabolism and an alternative AOX pathway for electron transport. Based on findings from this study, drugs targeting these pathways probably are effective against only intestinal *Cryptosporidium*, as gastric species such as *C. andersoni* and *C. muris* also use TCA cycle for energy metabolism and have a near conventional oxidative phosphorylation pathway for electron transport. Although *C. ubiquitum* has genomic features and gene content very similar to *C. parvum* and *C. hominis,* it may respond to drugs differently. For example, the absence of the AOX electron transport system in *C. ubiquitum* suggests that ascofuranone, which is a well-known AOX inhibitor in *T. brucei*, a protozoon relying on the AOX pathway for electron transport [29], may not be effective against this intestinal *Cryptosporidium* species. Similarly, although the synthesis and metabolism of isoprenoids have been targets for drug development in apicomplexan parasites [22, 47], the absence of the apicoplast in *Cryptosporidium* spp. and polyisoprenoid biosynthesis in *C. ubiquitum* indicates that effectiveness of this approach is probably limited against *Cryptosporidium* spp., especially *C. ubiquitum*. Currently, there is no specific anti-parasitic agent against *Cryptosporidium* spp. The diversity in mitochondrial metabolism and invasion-related proteins within the *Cryptosporidium* genus suggests that different approaches may be needed in the development of therapeutic agents against gastric and intestinal *Cryptosporidium* spp.

## Conclusions

In conclusion, we sequenced the genomes of six *C. ubiquitum* and *C. andersoni* isolates and performed a comparative genomics analysis of *Cryptosporidium* spp. and other apicomplexans. Results of this analysis suggest that *C. ubiquitum* and *C. andersoni* share many genomic features with *C. parvum* and *C. hominis*, but have divergent mitosome metabolism, electron transport chains, and invasion-related surface or secreted proteins and peptidases. This indicates the occurrence of a progressive reductive evolution in mitosome metabolism in the *Cryptosporidium* lineage within Apicomplexa. Associated with this is the differential evolution of invasion-related proteins, especially between the intestinal and gastric groups. With improved cultivation and animal models and development of genetic manipulation tools in recent years [48, 49], the biologic importance of these genetic differences among *Cryptosporidium* species could be verified. This in turn may lead to the development of intervention strategies against these important waterborne and zoonotic pathogens in both developing countries and industrialized nations.

## Methods

### Sample processing

*C. ubiquitum* isolates 39668, 39725, and 39726 were collected from sporadic cases of human cryptosporidiosis in Wisconsin, USA in summer, 2013, whereas *C. andersoni* isolates 30847, 31729, and 37034 were collected from beef cattle in Alberta, Canada in 2009, dairy cattle in Henan, China in 2010, and water buffalo in Kafr El Sheikh, Egypt in 2011, respectively. They were diagnosed to *Cryptosporidium* species by PCR-RFLP analysis and DNA sequencing of the small subunit rRNA gene [46]. *C. ubiquitum* was further subtyped by DNA sequence analysis of the gp60 gene [12]. Among the three *C. ubiquitum* isolates, 39668 and 39726 belonged to the XIIb subtype family whereas 39725 belonged to the XIIc subtype family. *Cryptosporidium* oocysts were purified from the specimens using a combination of sucrose and cesium chloride gradient centrifugation and immunomagnetic separation [50]. Total genomic DNA was extracted from purified oocysts using the QIAamp®DNA Mini Kit (Qiagen Sciences, Germantown, Maryland), after the oocysts were subjected to five freeze-thaw cycles and overnight digestion with proteinase K. Extracted DNA was amplified using REPLI-g Midi Kit (Qiagen).

### Genome sequencing and assembly

Genomes of *C. ubiquitum* and *C. andersoni* were sequenced on an Illumina Genome Analyzer IIX using the Illumina TruSeq (v3) library protocol, with 100 × 100 bp paired-end reads generated. Sequence reads with quality score below 30 were trimmed using CLC Genomics Workbench 7.03 (http://www.clcbio.com/products/clc-genomics-workbench). Using the same software, they were assembled *de novo* into contigs with word size of 40, bubble size of 50, and minimum contig length of 500 bp. Assemblies of *C. ubiquitum* isolate 39726 and *C. andersoni* isolate 30847 were selected for gene prediction, genome annotation and comparative genomics analysis.

### Analysis of genome structure

After the *de novo* assembly of sequencing reads, Mauve 2.3.1 [51] and MUMmer 3.2.3 [52] were used to map *C. ubiquitum* contigs to the published *C. parvum* IOWA genome [5] and *C. andersoni* contigs to the published *C. muris* RN66 genome (CryptoDB) using default parameters. Unmapped contigs represented *C. ubiquitum-* or *C. andersoni*-unique ones or those from contaminating microbes. BLASTN [53] analysis of GenBank nucleotides database was used to identify contigs from contaminants using e-value threshold as 1e-10. The syntenic relationship between the *C. ubiquitum* or *C. andersoni* genome and *C. parvum* genome was visualized by using Circos 0.67 [54], with sequence identities of >75% shown.

Software packages tRNAscan-SE 1.3.1 [55] and ARAGORN 1.2.36 [56] were used to identify tRNAs. Both of them were executed at the default settings using the general tRNA model or standard genetic codon. Ribosomal RNA genes were predicted using RNAmmer 1.2 [57] and BLASTN. Other genomic features such as mean length, N50 and N90 of contigs and GC content were calculated.

### Gene prediction

Protein-coding genes were predicted using a pipeline of three software packages, including AUGUSTUS 2.7 [58], SNAP [59], and GeneMark-ES [60]. All three software packages were run suing the default settings. Prior to their use in gene prediction, AUGUSTUS and SNAP were trained with the gene model of the published *C. parvum* IOWA genome (CryptoDB release-6.0) for the prediction of *C. ubiquitum* genes, and gene model of the published *C. muris* RN66 genome (CryptoDB release-6.0) for the prediction of *C. andersoni* genes. The length and identities of gene sequences predicted by the three approaches were compared with the reference genomes. As the outcome from AUGUSTUS agreed mostly with the reference genomes, the gene set predicted by AUGUSTUS was supplemented with new genes predicted by GeneMark-ES and SNAP. Only genes predicted by both software package were added to the proteome predicted by AUGUSTUS.

The frequency of protein translation codon usage for each genome was calculated using INCA 2.1 [61]. The entire coding region and 500 bp upstream were extracted to search the most conserved or overrepresented motifs in each genome by using MEME 4.9 [62] with the width threshold as 6-8 bp. SignalP 4.1 [63] and TMHMM 2.0 [64] were used to identify signal peptides and the transmembrane domains in predicted proteins, respectively. Proteins with GPI anchor sites were identified by using the GPI-SOM webserver [65]. The mucin-type O-glycoproteins were predicted by using NetOGlyc 3.1 [66]. These software packages were run using the default parameters.

### Functional annotation and comparative genomics analysis proteins

The predicted proteomes of *C. ubiquitum* and *C. andersoni* were compared with those of other *Cryptosporidium* spp. using OrthoMCL [67], BLASTP [53] and in-house scripts to identify shared orthologs and potential species-unique proteins in each genome. OrthoMCL and BLASTP were run with e-value thresholds of 1e-1 and 1e-3, respectively. The in-house scripts were used to extract synthenic genes located in the genome but not identified by OrthoMCL or BLASTP. The genes located in the non-syntenic regions were considered as potential species-unique protein-encoding genes. Protein domains in them were identified using Pfam (http://pfam.xfam.org/) with the default setting [68].

Comparative metabolism analyses of *C. ubiquitum* and *C. andersoni* were performed using the web server KAAS [69] with the BBH (Bi-directional Best Hit) method and eukaryote gene model. BLASTP search of the GenBank NR database was used as a supplemental analysis. Data on metabolic pathways, catalytic enzymes, and functional proteins of other apicomplexans were retrieved primarily from online databases LAMP (Library of Apicomplexan Metabolic Pathways, release-2) [21], KEGG (http://www.genome.jp/kegg/), and Pfam [68], with the EuPathDB (http://eupathdb.org/eupathdb/) as the supplement. Comparisons of transporter proteins and invasion-related proteins among *Cryptosporidium* species were mainly based on Pfam search results.

### Phylogenetic analysis

A venn diagram of shared orthologs and species-specific genes of *C. parvum*, *C. hominis*, *C. ubiquitum* and *C. andersoni* was drawn using the web-tool Venny (http://bioinfogp.cnb.csic.es/tools/venny/). The amino acids sequences of 100 orthologs shared by *C. ubiquitum*, *C. andersoni* and other common apicomplexan parasites, including *C. parvum, C. hominis*, *Eimeria tenella*, *Neospora caninum*, *T. gondii*, *P. falciparum*, *P. vivax*, *Babesia bovis*, *Theileria annulata*, and *T. parva*, were extracted to construct a neighbor-joining tree with the maximum composite likelihood mode for distance calculation and 1000 replications for bootstrapping. The concatenated amino acid sequences were aligned with ClustalX [70], trimmed off un-aligned region using Gblocks [71], and used in MEGA 6 [72] for the construction of the neighbor-joining tree. A concatenated sequence from the free-living photosynthetic chromerid, *Chromera velia*, was used to root the tree.

## Additional files

Additional file 1: Table 1. Summary of *Cryptosporidium ubiquitum* and *C. andersoni* genomes sequenced in this study. (DOCX 19 kb)
Additional file 2: Figure S1. A) Similarity in codon usage frequency among *Cryptosporidium parvum*, *C. ubiquitum* and *C. andersoni*. As expected, the third position of the most commonly used codons mostly has A or T, except for the UGG codon for tryptophan and UTG codon for methionine. B) The most over-represented sequence motifs in upstream regions of protein-encoding genes of *C. parvum, C. ubiquitum* and *C. andersoni*. The E2F-like motif, 5′-TGGCGCCA-3′, is the dominant one in all *Cryptosporidium* species. (DOCX 429 kb)
Additional file 3: Figure S2. A) Syntenic relationship between the genomes of *Cryptosporidium parvum* and *C. ubiquitum* or *C. andersoni*. Syntenic sequences (identity >75%) are connected with lines. The colors of lines represent different chromosomes of *C. parvum*. B) Venn diagram of shared orthologs and species-specific genes among four *Cryptosporidium* species. Because of the use of different gene prediction approaches in genome annotation, species-specific genes are generally over-estimated. (DOCX 2376 kb)
Additional file 4: Table S2. Orthologs of potential invasion-related mucin-type glycoproteins, thrombospondin-related adhesive proteins (TRAPs), insulinase-like proteases, secreted MEDLE family proteins, and rhomboid-like proteases of *Cryptosporidium* spp. (DOCX 21 kb)
Additional file 5: Table S3. Sequences of putative *Cryptosporidium ubiquitum-* and *C. andersoni*-specific mucin-type glycoproteins. O-linked glycosylation sites (indicated by single-letter amino acid codes) are shown in the second sequence of each mucin-type glycoprotein. (DOCX 27 kb)

## Abbreviations

AOX: Alternative oxidase
DMAPP: Dimethylallyl diphosphate
EGF: Epidermal growth factor
FPP: Farnesyl diphosphate
GPI: Glycosylphosphatidylinositol
IPP: Isopentenyl diphosphate
MQO: Malate:quinone oxidoreductase
OGDH: Oxoglutarate dehydrogenase complex
PDH: Dehydrogenase complex
PNO: Pyruvate:NADP+ oxidoreductase
PPP: Polyprenyl diphosphate
PVM: Parasitophorous vacuole membrane
TCA: Tricarboxylic acid
TPP: Thiamine pyrophosphate
TRAP: Thrombospondin-related adhesive proteins
TSP: Thrombospondin
VWA: Willebrand factor type A
