# Supplementary material for: Evolution of mitosome metabolism and invasion-related proteins in Cryptosporidium
Source: BMC Genomics. 2016 Dec 8;17:1006. doi: 10.1186/s12864-016-3343-5 (PMC5146892; doi:10.1186/s12864-016-3343-5)
Supplement: Additional file 1: Table 1. — Summary of Cryptosporidium ubiquitum and C. andersoni genomes sequenced in this study. (DOCX 19 kb) [file 12864_2016_3343_MOESM1_ESM.docx]

**Additional file 1: Table S1:** Summary of genomes of *Cryptosporidium ubiquitum* and *C. andersoni*.

| Species | *C. ubiquitum* | | | *C. andersoni* | | |
| --- | --- | --- | --- | --- | --- | --- |
| Isolates | 39726 | 39668 | 39725* | 30847 | 31729 | 37034 |
| Host source | Human | Human | Human | Cattle | Cattle | Cattle |
| Source location | USA | USA | USA | Canada | China | Egypt |
| Total sequencing reads | 137,998,120 | 125,216,952 | 123,109,262 | 27,157,694 | 12,774,228 | 21,539,030 |
| Total length of assembly (bp) | 9,133,657 | 9,847,091 | 12,132,003 | 9,566,194 | 9,774,526 | 14,076,035 |
| No. of contigs | 143 | 718 | 1,335 | 313 | 559 | 3,788 |
| Size of draft genome (bp) | 8,971,871 | 8,965,564 | 9,100,863 | 9,097,995 | 9,082,595 | 8,965,616 |
| No. of contigs in draft genome | 27 | 27 | 276 | 96 | 278 | 1,438 |
| Maximum contig size (bp) | 1,292,350 | 1,292,604 | 278,257 | 586,771 | 434,208 | 63,770 |
| Mean length (bp) | 332,292 | 332,058 | 32,974 | 94,771 | 32,671 | 6,235 |
| N50 (bp) | 775,861 | 676,938 | 82,501 | 253,055 | 77,269 | 12,327 |
| N90 (bp) | 196,338 | 274,149 | 23,275 | 76,460 | 20,368 | 2,798 |

*39725 appears to be a mixed infection with another minor population related to *Cryptosporidium* chipmunk genotype I (skunk genotype?).
