# Supplementary material for: Evolution of mitosome metabolism and invasion-related proteins in Cryptosporidium
Source: BMC Genomics. 2016 Dec 8;17:1006. doi: 10.1186/s12864-016-3343-5 (PMC5146892; doi:10.1186/s12864-016-3343-5)

**Additional file 2: Figure S1:** A) Codon usage bias in *Cryptosporidium parvum*, *C. ubiquitum* and *C. andersoni*. All three *Cryptosporidium* species have a similar codon usage frequency. As expected, the third position of the most commonly used codons mostly has A or T, except for the UGG codon for tryptophan and UTG codon for methionine. B) The most over-represented sequence motifs in upstream regions of protein-encoding genes of *C. parvum, C. ubiquitum* and *C. andersoni*. The E2F-like motif, 5′-TGGCGCCA-3′, is dominant in all *Cryptosporidium* species.


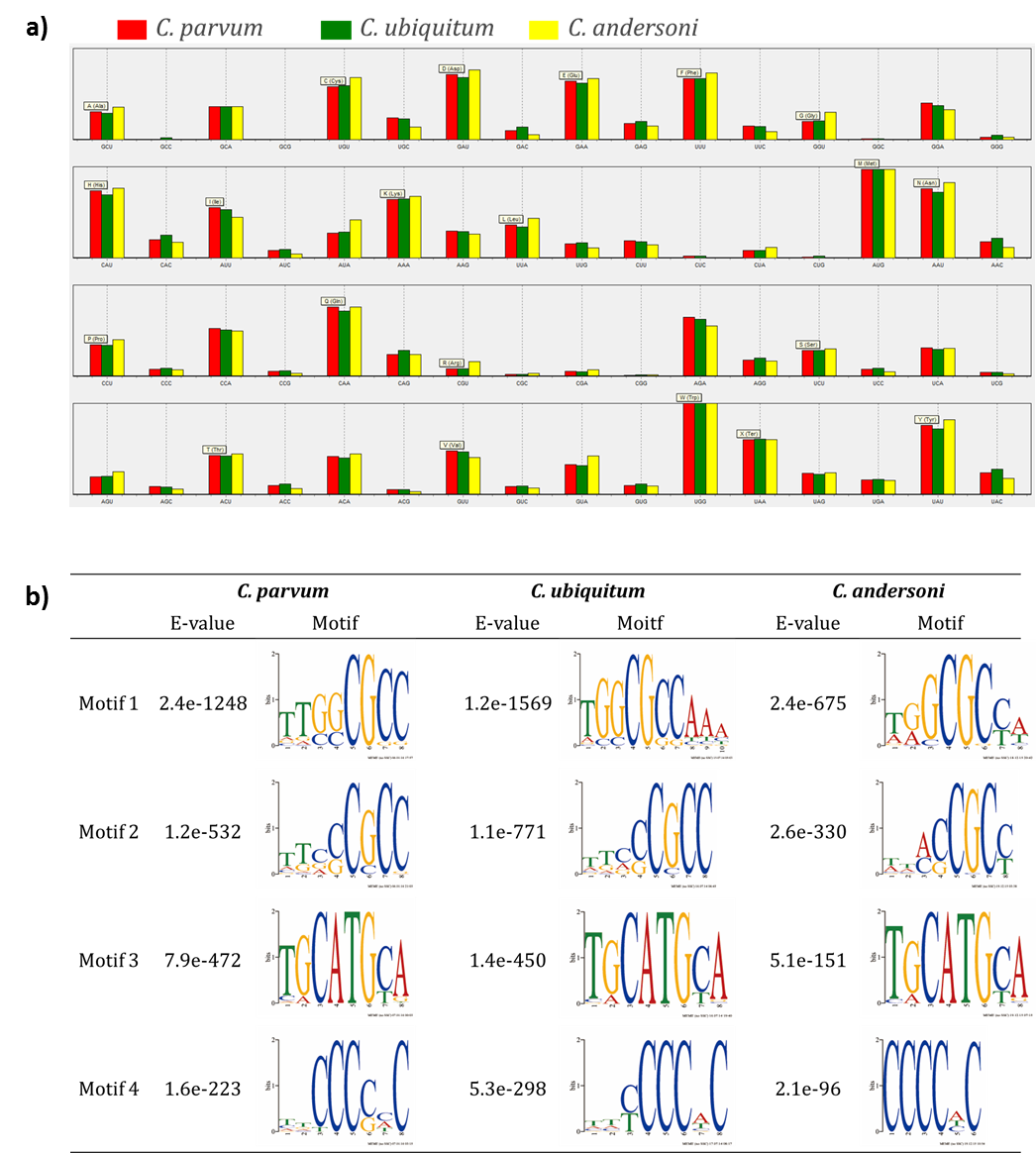

Supplement: Additional file 2: Figure S1. — A) Similarity in codon usage frequency among Cryptosporidium parvum, C. ubiquitum and C. andersoni. As expected, the third position of the most commonly used codons mostly has A or T, except for the UGG codon for tryptophan and UTG codon for methionine. B) The most over-represented sequence motifs in upstream regions of protein-encoding genes of C. parvum, C. ubiquitum and C. andersoni. The E2F-like motif, 5′-TGGCGCCA-3′, is the dominant one in all Cryptosporidium species. (DOCX 429 kb) [file 12864_2016_3343_MOESM2_ESM.docx]
