# Supplementary material for: Evolution of mitosome metabolism and invasion-related proteins in Cryptosporidium
Source: BMC Genomics. 2016 Dec 8;17:1006. doi: 10.1186/s12864-016-3343-5 (PMC5146892; doi:10.1186/s12864-016-3343-5)

**Additional file 3: Figure S2:** A) Syntenic relationship between the genomes of *Cryptosporidium parvum* and *C. ubiquitum* or *C. andersoni*. Syntenic sequences (identity >75%) are connected with lines. The colors of lines represent different chromosomes of *C. parvum*. B) Venn diagram of shared orthologs and species-specific genes among four *Cryptosporidium* species. Because of the use of different gene prediction approaches in genome annotation, species-specific genes are generally over-estimated.


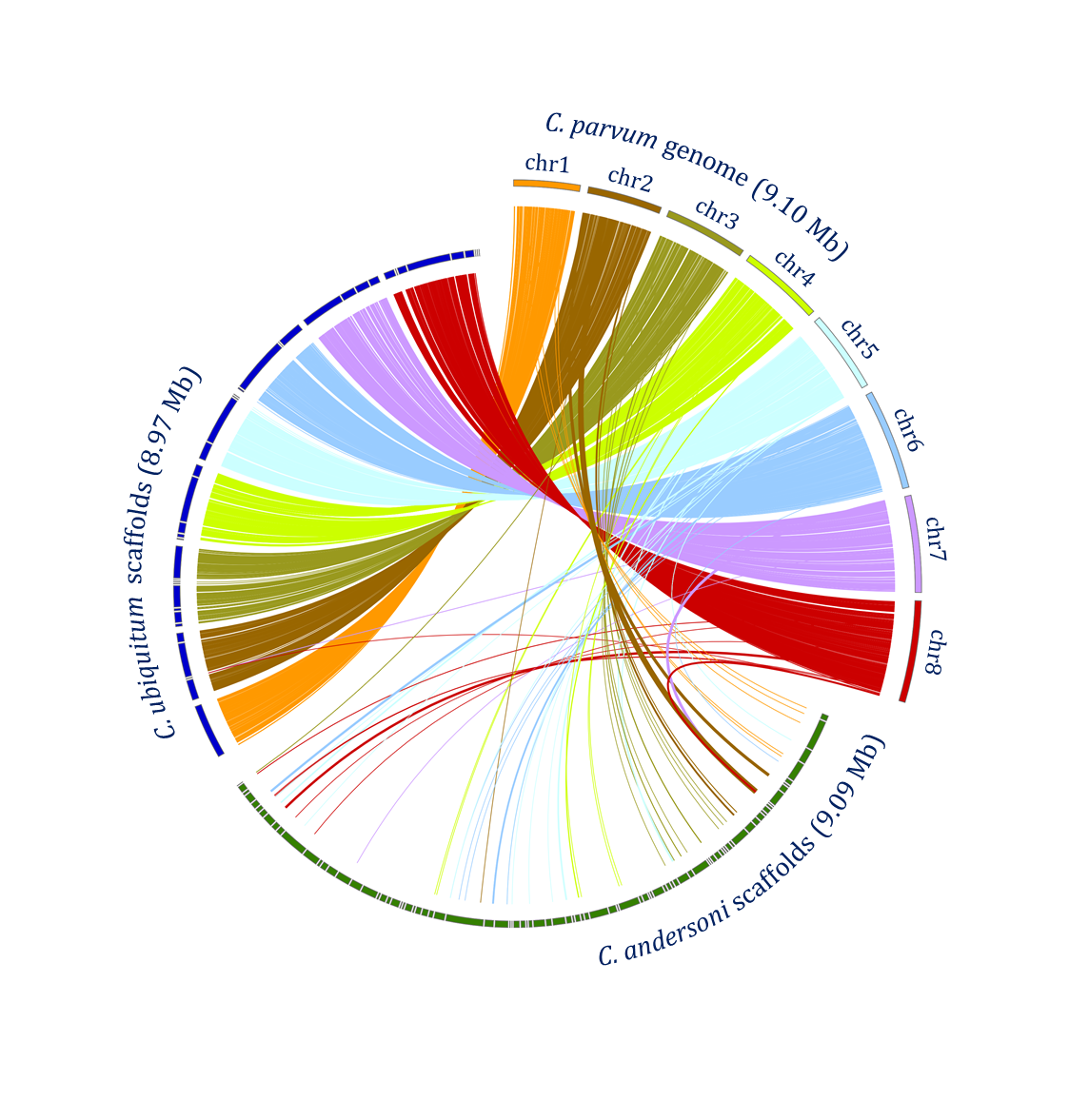


**A**

**B**


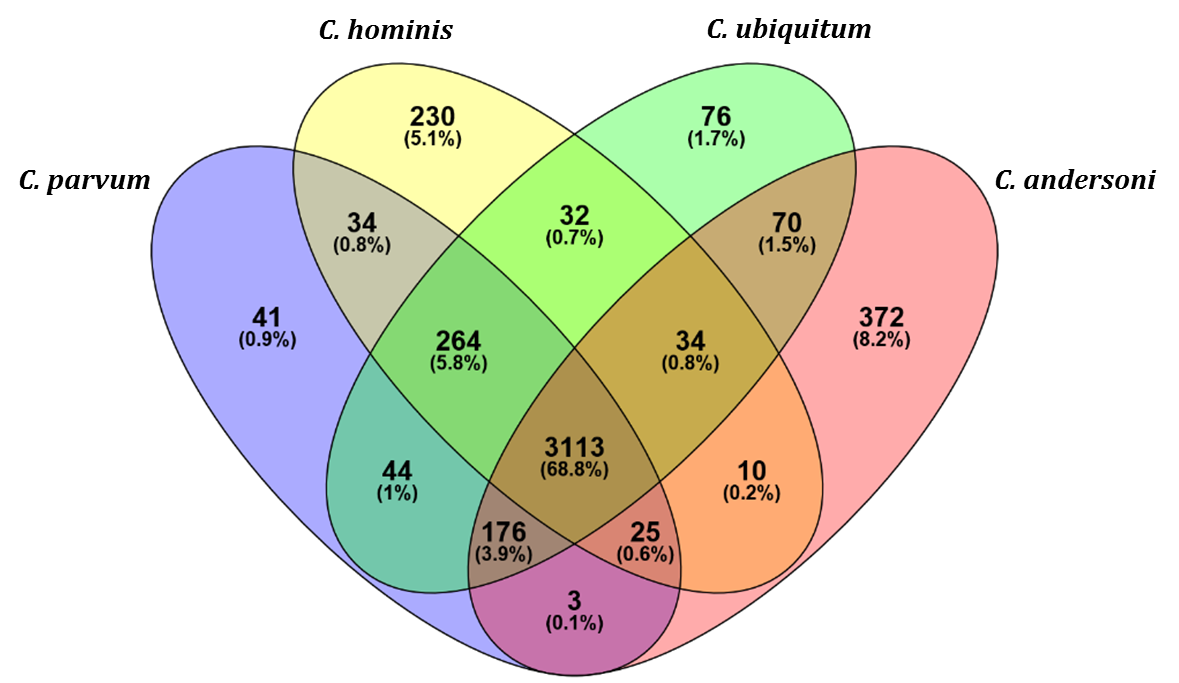

Supplement: Additional file 3: Figure S2. — A) Syntenic relationship between the genomes of Cryptosporidium parvum and C. ubiquitum or C. andersoni. Syntenic sequences (identity >75%) are connected with lines. The colors of lines represent different chromosomes of C. parvum. B) Venn diagram of shared orthologs and species-specific genes among four Cryptosporidium species. Because of the use of different gene prediction approaches in genome annotation, species-specific genes are generally over-estimated. (DOCX 2376 kb) [file 12864_2016_3343_MOESM3_ESM.docx]
