# Supplementary material for: Evolution of mitosome metabolism and invasion-related proteins in Cryptosporidium
Source: BMC Genomics. 2016 Dec 8;17:1006. doi: 10.1186/s12864-016-3343-5 (PMC5146892; doi:10.1186/s12864-016-3343-5)
Supplement: Additional file 4: Table S2. — Orthologs of potential invasion-related mucin-type glycoproteins, thrombospondin-related adhesive proteins (TRAPs), insulinase-like proteases, secreted MEDLE family proteins, and rhomboid-like proteases of Cryptosporidium spp. (DOCX 21 kb) [file 12864_2016_3343_MOESM4_ESM.docx]

**Additional file 4: Table S2:** Orthologs of potential invasion-related mucin-type glycoproteins, thrombospondin-related adhesive proteins (TRAPs), insulinase-like proteases, secreted MEDLE family proteins, and rhomboid-like proteases of *Cryptosporidium* spp.

| **Family** | **Protein** | ***C. parvum*** | ***C. hominis*** | ***C. ubiquitum*** | ***C. andersoni*** |
| --- | --- | --- | --- | --- | --- |
| **Adhesin (mucin-type glycoprotein)** | gp900 | cgd7_4020 | Chro.70447 | cubi03073 | cand012040 |
|  | gp60/40/15 | cgd6_1080 | Chro.60138 | cubi02234 | absent |
|  | P23 | cgd4_3620 | Chro.40414 | cubi01522 | absent |
|  | CP2 | cgd6_5410 | Chro.60623 | cubi02667 | absent |
|  | Clec | cgd3_440 | Chro.30061 | cubi00854 | cand034120 |
|  | Muc1 | cgd2_390 | Chro.20046 | cubi00389 | absent |
|  | Muc2 | cgd2_400 | Chro.20047 | cubi00388 | absent |
|  | Muc3 | cgd2_410 | Chro.20048 | cubi00387 | absent |
|  | Muc4 | cgd2_420 | Chro.20049 | cubi00386 | absent |
|  | Muc5 | cgd2_430 | Chro.20050 | cubi00385 | absent |
|  | Muc6 | cgd2_440 | Chro.20051 | absent | absent |
|  | Muc7 | cgd2_450 | Chro.20052 | cubi00384 | absent |
|  | Muc8 | cgd1_470 | Chro.10057 | cubi00043 | absent |
|  | Muc9 | cgd1_3550 | Chro.10397 | cubi00351 | cand017610 |
|  | Muc10 | cgd2_3290 | Chro.20345 | cubi00739 | cand002970 |
|  | Muc11 | cgd3_720 | Chro.30096 | cubi00884 | cand033810 |
|  | Muc12 | cgd4_1300 | Chro.40149 | cubi01576 | absent |
|  | Muc13 | cgd5_340 | Chro.50359 | cubi01726 | cand013850 |
|  | Muc14 | cgd5_1210 | Chro.50263 | cubi01812 | absent |
|  | Muc15 | cgd5_2060 | Chro.50173 | cubi01892 | cand035520 |
|  | Muc16 | cgd6_710 | Chro.60092 | cubi02197 | cand024260 |
|  | Muc17 | cgd6_5400 | Chro.60622 | cubi02666 | absent |
|  | Muc18 | cgd7_4660 | Chro.70514 | cubi03137 | cand012700 |
|  | Muc19 | cgd8_410 | Chro.80053 | cubi03264 | cand021110 |
|  | Muc20 | cgd8_700 | Chro.80087 | cubi03294 | absent |
|  | Muc21 | cgd8_1160 | Chro.80136 | cubi03340 | absent |
|  | Muc22 | cgd8_2800 | Chro.80327 | cubi03505 | cand023600 |
|  | Muc23 | cgd8_3520 | Chro.80404 | cubi03581 | cand004650 |
|  | Muc24 | cgd8_4830 | Chro.80555 | cubi03710 | cand009540 |
| **TRAP** | TRAP-C1(TSP1) | cgd1_3500 | Chro.10390 | cubi00344 | cand017670 |
|  | TRAP-C2(TSP2) | cgd5_3420 | Chro.50029 | cubi02109 | cand013510 |
|  | TSP3 | cgd1_3510 | Chro.10391 | cubi00345 | cand017660 |
|  | TSP4 | cgd8_150 | Chro.80025 | cubi03237 | cand020810 |
|  | TSP5 | cgd6_1300 | Chro.60164 | cubi02255 | cand007140 |
|  | TSP6 | cgd6_2310 | Chro.60270 | cubi02355 | cand006020 |
|  | TSP7 | cgd5_4470 | Chro.60103 | cubi01995 | cand034510 |
|  | MIC1(TSP8) | cgd6_780 | Chro.60102 | cubi02205 | cand024180 |
|  | TSP9 | cgd6_800 | Chro.60104 | cubi02206 | cand024170 |
|  | TSP10 | cgd2_3080 | Chro.20323 | cubi00687 | cand026770 |
|  | TSP11 | cgd6_1660 | Chro.60203 | absent | cand006680 |
|  | TSP12 | cgd8_540 | Chro.80069 | cubi03279 | cand021260 |
| **Insulinase-like protease** | INS1 | cgd1_1680 | Chro.10193 | cubi00162 | cand032340 |
|  | INS2 | cgd1_3840 | Chro.10431 | cubi00379 | absent |
|  | INS3 | cgd2_920 | Chro.20103 | cubi00472 | absent |
|  | INS4 | cgd2_930 | Chro.20104 | cubi00473 | cand007890 |
|  | INS5 | cgd2_2760 | Chro.20292 | cubi00656 | cand003730 |
|  | INS6 | cgd2_4270 | Chro.20459 | cubi00802 | cand002300 |
|  | INS7 | cgd3_4170 | Chro.30468 | cubi01223 | absent |
|  | INS8 | cgd3_4180 | Chro.30469 | cubi01224 | absent |
|  | INS9 | cgd3_4190 | Chro.30470 | cubi01225 | absent |
|  | INS10 | cgd3_4200 | absent* | cubi01227 | absent |
|  | INS11 | cgd3_4210 | Chro.30474 | cubi01228 | cand003500 |
|  | INS12 | cgd3_4220 | Chro.30475 | cubi01229 | cand003510** |
|  | INS13 | cgd3_4240 | absent* | cubi01231 | cand027870** |
|  | INS14 | cgd3_4250 | Chro.30478 | cubi01232 | cand027880** |
|  | INS15 | cgd3_4260 | Chro.30479 | cubi01233 | cand026930 |
|  | INS16 | cgd3_4270 | Chro.30480 | absent | cand003460 |
|  | INS17 | cgd3_4280 | Chro.30482 | cubi01234 | absent |
|  | INS18 | cgd4_4240 | Chro.40483 | cubi01672 | absent |
|  | INS19 | cgd6_5510 | absent | absent | absent |
|  | INS20 | cgd6_5520 | absent | absent | absent |
|  | INS21 | cgd7_2080 | Chro.70239 | cubi02884 | cand030190 |
|  | INS22 | cgd8_2720 | Chro.80317 | cubi03496 | cand023510 |
| **Secreted MEDLE family protein** | MEDLE1 | cgd5_4580 | absent | absent | absent |
|  | MEDLE2 | cgd5_4590 | absent | absent | absent |
|  | MEDLE3 | cgd5_4600 | Chro.50507 | absent | absent |
|  | MEDLE4 | cgd5_4610 | absent | absent | absent |
|  | MEDLE5 | cgd6_5480 | absent | absent | absent |
|  | MEDLE6 | cgd6_5490 | absent | absent | absent |
| **Rhomboid-like protease** | ROM1 | cgd3_980 | Chro.30129 | cubi00912 | cand038380 |
|  | ROM2 | cgd6_760 | Chro.60098 | cubi02202 | cand024210 |
|  | ROM3 | absent | absent | absent | cand007640 |
|  | ROM4 | cgd7_3020 | Chro.70339 | cubi02974 | cand019770 |

* The absence of some genes from *C. hominis* genome may be caused by segmented small contigs;

** These coding genes are not orthologous with those from other *Cryptosporidium* spp.
