# Supplementary material for: Evolution of mitosome metabolism and invasion-related proteins in Cryptosporidium
Source: BMC Genomics. 2016 Dec 8;17:1006. doi: 10.1186/s12864-016-3343-5 (PMC5146892; doi:10.1186/s12864-016-3343-5)
Supplement: Additional file 5: Table S3. — Sequences of putative Cryptosporidium ubiquitum- and C. andersoni-specific mucin-type glycoproteins. O-linked glycosylation sites (indicated by single-letter amino acid codes) are shown in the second sequence of each mucin-type glycoprotein. (DOCX 27 kb) [file 12864_2016_3343_MOESM5_ESM.docx]

**Additional file 5: Table S3:** Sequences of putative *Cryptosporidium ubiquitum-* and *C. andersoni*-specific mucin-type glycoproteins. O-linked glycosylation sites (indicated by amino acids) are shown in the second sequence of each mucin-type glycoprotein.

| **Putative *C. ubiquitum*-specific mucin-type glycoproteins (n=7)** | |
| --- | --- |
| Name: cubi03712 | Length: 68 |
| MKDLDSNNYGRPHFNLNLVVVVVVVVEVEVSGSSSGSSSGSSSGRGSSSGGSSDRGGSGGGGSGGGGS | |
| ................................SSS.SSS.SSS...SSS..SS....S....S....S | |
|  |  |
| Name: cubi03766 | Length: 724 |
| MVNQEVQELLKFQKVQMERKLQKHQQQPSPLQPQPPPLQPQPSPLQPQPSSLQPQPPPSSFPPPPSPSSPPSIQPEPEPL | |
| PLQPQPSPSPSPPSLQPKSQTSPLQPQTSPSQPSPPPSPPPPPLPPSLSPQSPLSPLPSQPSPLHSQSPIPPSPSVSLPS | |
| FSPYQSPNPSTPSSPPLPPPPPPPPSSPLPPPPSSPLPPPPPSSPLPPPPSSSPLPPPPSSSPLPPPPPSPLPPPPSSPL | |
| PPPPSPKPPLPPASKIPPPVPPKTYKYAKLITSEISGLTDNQSNDLSDDQTEDEQNHLKTTQKQTNRLNTTQIQNLMKLQ | |
| NQIIMKSFLRENGLEDLYLLNCNLNTLDRIYYLLEKVKKIILQVQVRETKMYKTNLNSFITSHSIMKKNIETTNYQTLVN | |
| SLNVLKDYLKQILDDCKSLHNIQPSTEIHTEHYSGKIDECENEKYIPLIDLYSFLFKNYKKLIANSTFGARMLNHFVTIL | |
| KDFTEFNNCFVQEKIEYWKSLLQEYKKNIKYEIKMDNKTKCSGYSHHGVMCSINECIKTSEDRLKSEKIIVYKFMVKLLR | |
| ELISKCEQVKNDPNFDDNLKGQLFNENCIYKYNIRYNIQSKFLNNSNQNQVLERIQNLIQSFGGIVFIHDSRDCNPNNIV | |
| KILEFIARLKVIKSLGDNNTNLYSSTITNQEEIKALNIKSINFLIKLMLDMISFCWTYGIYKKNDYIQRSDTKLIDHWVN | |
| FKPY |  |
| ..........................................S......SS.......SS.....S.SS..S........ | |
| ......S.S.S..S....S.TS.....TS.S..S...S........S.S..S..S...S..S...S.S....S.S.S..S | |
| .S...S...ST.SS...........SS......SS.......SS......SSS......SSS.......S......SS.. | |
| ....S........S.........T........................................................ | |
| ................................................................................ | |
| ................................................................................ | |
| ................................................................................ | |
| ................................................................................ | |
| ................................................................................ | |
| .... |  |
|  |  |
| Name: cubi03293 | Length: 954 |
| MMKDAQSGSSSMNSDINFFKSLNIVYHGIVKYCHRFGILNLYKSLENKQEPRFETVEWSSYPQLMDFIFPQFPLEDRMMT | |
| DYQLFGFDFLLKSGSYSYFFDISTGKMTLLDDFGGGSEPGEEGKSKLISDQPLITEEVPIQIENEEDDDFLLTVEIPGTT | |
| PEQEIVDNLDLYSLLEHDFSSNVIAFSEELTSLLLPKHVEAAIQYLQGCNIKMLFIINSRIETVKELLNRLNLLSTGING | |
| IEGDLLMKDAISNVLSNLLKKLQSYLEKCRNINKYKVPWIHIAGLKGEKATILQSLHPSFVDSSIIPFYCDNVDLKDVLE | |
| FIKNDKLKIKSLKEGLNKMKNHNKNKSRLKKELLILKASTRRNTLLFEICDDFNNLKYSDTGLYYKVLSVLSDFESNMKV | |
| RESNIKEFYSKMGLPFKSESAIDQLKNTEMDDEEELNTLYGIYLSLANGANPLTMEEIESLANANKFSKIDYLKEIIQSL | |
| ERKEEENDVNKKTQESDEENIDLVNILTSDEEILSSYSLYSSIAQNPLNLIEIVEISDYDKTKVLSLLAKLNQDLLGENQ | |
| SNISEKPGGGDGPGGAGEEEGAVGGVGIENEHLLKDEEILNKYLLYRSLVLDKNTPLELYDLIAIPGNDKKENLMQTLDE | |
| AIAMMNKDDKGSEKSLLPKKLTEPSPLPPQEESATGRPKKPSYPLASSILSPSGTTGGADGRRDPGARPKQLLPKPTLKT | |
| NVVTQQELRLDENLLNDLEVYTKYLDYSAVAQNPLNLEDLVLASNNNKIELIKMLKEAISQANIPPMFPTSPLPPAPVPP | |
| TPLPPTPVPAPLPPAPVPKPLPPAPSQPMSPSVGPKIQLKFTNGKLDQNSVDQSKLSLSTIQKFSKLVQVGCTIESLNEL | |
| QFLLNVSRADLAHLKALEKFVKGPAARCSTNRSSCNNCFVCNSRKSIDKDITNMEKVIAALQALVGYCIQNMKK | |
| ................................................................................ | |
| ................................................................................ | |
| ................................................................................ | |
| ................................................................................ | |
| ................................................................................ | |
| ................................................................................ | |
| ................................................................................ | |
| ................................................................................ | |
| ..................................T............................................. | |
| .....................................................................TS......... | |
| T....T.......................................................................... | |
| .......................................................................... | |
|  |  |
| Name: cubi02672 | Length: 251 |
| MFKEINSILLFICITSIFNFYKGEVEQLAPATKNLSFLKLKNGIYGRSTRSGGGRRGGASARFGNRAPQTQEGQGPLQFP | |
| PPDYPEAETDPLQYPPPDYPGAETDSLQYPPPDYPGAQSLLKYPPPDYLETQGPLQYPPTDYEETVESRSPRPSRPHHRP | |
| SNRDNRSPSTRNVGTPLGFNQHSNPNTGGNRGPRTSHRAVAAPPPPPPPPPPPPPPPPSRQDPRAGVGNQSLASQLAQGG | |
| ARLRKTGVKLT |  |
| .....................................................................T.......... | |
| ........T..............T..........................T........T....T..S.....S...... | |
| S........T........................TS......................S..................... | |
| ........... |  |
|  |  |
| Name: cubi02033 | Length: 78 |
| MVTLGFYTSHTQSRRRRKERSRPGRTCTQGTPQSVPQTRVRTKEASVSLALLLNDTLVLLEGWGLTREGQRVCCTSRX | |
| ....................S....T.T..T..S...T........................................ | |
|  |  |
| Name: cubi00381 | Length: 493 |
| MNSFTYSVLTFIFIKLLGLWDFLVIHDIGYLDNGTNYIQPLCSSFIKLKGTNENDDLNNNKSAGTSRKGLFGLQGRSNLP | |
| SRRPYSGIFSSSGSLQHSRQTPFPQRTPYPRQTPFPQRTPFPQRTPFPQRTPRPLQTPPRHLPKRVFPQVMATPSLPTIP | |
| ESPEPTSSSESSRPSSRTSSGSSSPGSSRPSSPGSSRSPNPLTSKSSSLTQFSRSTPKPAPPRTPYSGDLLTMGYRSALD | |
| SIFGSKQSGSAGSSTPGQQRLSPESHGPQSSGGSRSPSPKSGKSQTPGVYTTTLKVELNKTPGTTRPNPKLTASKIEIAS | |
| KQSSSGKPSSSPEGFGSQRFRKSRSQSPKTPRSHSPKSGKEQTPGVYTSTLKVNLNQPPRTTRPNPKLTASKIKIASKQS | |
| NSGQQSRTPDPRQQHPTPRYQAPPSSSRSGVSTQGSTGRGYLASRCPLGDPCVRCLEPNRDNTLICATCNNFMKHEECDP | |
| PRHVSRIVSKKYK |  |
| ................................................................................ | |
| ....................T.....T.....T.....T.....T.....T.....T...............T.S..T.. | |
| .S...TSSS.SS..SS.TSS.SSS..SS..SS..SS.S....TS.SSS.T..S.ST........................ | |
| ..............T......S..S....SS..S.S.S..S....T....TTT........................... | |
| .............................T.................................................. | |
| .......T........T.......S.......T...T........................................... | |
| ............. |  |
|  |  |
| Name: cubi03765 | Length: 618 |
| MPGTSDSTEIPGEPGKPTMPGTSDSTEIPGESGKPAMPGTSDSTEIPGESGKPAIPGTSDSTEIPGESGKPAIPGTSEST | |
| EIPGESGKPAITGTSDSTKIPATPGTPEELETGAFPVPPKIGTEISPIPSPVPPKTYTNLNIDEKYIHPTAPETPEVTDD | |
| NTGNLKKDQTHEYTQQESDDDLSDVQSEDEQSHLKTTQKQTYRLTTTQIQNLMILQTQIRINDILKENGLDNLNLINCDL | |
| NTVNRIYELIKKAEQIISEIQIRKSSIDQIDLNSFITSHSVIKKNTETTNYQTLISSLNVLKDYLKQILDDCKSLYNIQP | |
| STEIHSKYYSSIIDECENEKYIPLIDLYSFFLKYYKRLMVNSTFGARMLNHFLTILINLNEIKQCLDEQKINSWKLLKEE | |
| YQNNLKEETKKATEIQLNGYSDHVAGSSINQSINNSEIHLINEKIKVYKIIIRLLNELILKCQELKNNPDLETNSKIHVF | |
| NENCIYKYNIKESMIRFKLFNGLNMSQRDEILERIQRLIQSLGGFISIYDSKDCNPNNIVKILESIARLKTIKDIEKNNT | |
| NLYENLKENKILNLNLRSIKFLIKLMIDMISFCWTYVNMNKIVKLKLIIKHWVNFKPY | |
| ...TS.ST.........T...TS.ST.....S.......TS.ST.....S.......TS.ST.....S.......TS.ST | |
| .....S.....T.TS.ST....T..T.....T..........T..............T...................... | |
| ................................................................................ | |
| ................................................................................ | |
| ................................................................................ | |
| ................................................................................ | |
| ................................................................................ | |
| .......................................................... | |

| **Putative *C. andersoni*-specific mucin-type glycoproteins (n=30)** | |
| --- | --- |
| Name: cand003570 | Length: 102 |
| TETTTKPTTHTTQTTTIPATHSTKAITNPTTHSTSTSMESSTSYSSNDILSQGQIAGIVSGAVAGGAVGGALLTAGGFLA | |
| ANSQNSSNNRRFLRGSMIYALX | |
| T.TTT..TT.TT.TTT...T.ST...T..TT.STSTS..SSTS.S................................... | |
| ...................... |  |
|  |  |
| Name: cand007060 | Length: 191 |
| MTSLNTYSSPRSTGRLSPRAIIEKRQVKEHKSPRKLAAEHVIYSPIFHQGISSAHHISSNIKNLKSPKSIKSGTHAKSSP | |
| LKHHTSHHANHDINHYSKSPTRRSLRLLMESTHNKHNTHILHHQITNNSYQTANLAEKRSSQIKSLVTLKKNKKKNITSP | |
| KKSKATNKVSTKKRISKSSKSSARVSPKKFX | |
| .TS..T.S........................................................................ | |
| ................................................................................ | |
| ..........T.................... | |
|  |  |
| Name: cand002210 | Length: 355 |
| MTIRIFIYLIILFYLISKINCSDTSPINSKFCVATPYIFDKRCLAIFLEVYNLNFEVFSLSKRFNWILSEQAMLINADLF | |
| CNLRKMYAYLHKASRLYGSMDKFQELRVTNPFECSIGPLNNPTSILLENNLYRIKAEVDGLECACRCLRAQIKILEEINV | |
| GYCIFILEFILFILERAYFLLTVLEDILGFLQNILFYYFGVGDNSYINTHRLEVLLEVPGIIQDELPGNEIISILINYIS | |
| TGDSFDCYSKMYYDADLSEFESRISRSVYLQHNMMCENEIKSPCNSDDQDSNICTSQKVDLVSSKVEFPESRNEEVIKKE | |
| VSLSPDSSHRRGRSRRSRSSSSSSSSHRNRISSSS | |
| ................................................................................ | |
| ................................................................................ | |
| ................................................................................ | |
| ................................................................................ | |
| ..................SS.....S.....SSSS | |
|  |  |
| Name: cand003220 | Length: 271 |
| MKSNLISNYLIFLLFYIFLLKIKNVKSSTDSSSINDTISDLDINLLSMEDIFLLCNNFGYLLDNEKSKIYNLLISYKNYY | |
| HFVEDESRINECIQKLQSEISVESEINFKSDPMKLLRIPVKMPKLFVFEVQPISQLPIQPLKYVAKSIQVSNIREEDITI | |
| PLKDIDMRESEDYEENNTITDTLSDIKTELSISNNSEDDQSGNNYGEIDSEGSYILDNSDNSTLSLPSLPSLPSIPSIPS | |
| IPYNTDHSSALDSLRSSSIYSPTLTTDISDX | |
| ................................................................................ | |
| ................................................................................ | |
| ..............................................................T..........S..S... | |
| ....T..S..............T.TT..S.. | |
|  |  |
| Name: cand009790 | Length: 298 |
| MILKINISLIIFILFINNSLSREGKKPRFPENQNDKLNNSSMRSNCSDSYHNYSCSFLRNISMSSSPGIYSVTSVYKSTT | |
| PTISRTLKISGTSKIPELSEMSDLSETSDMLEIPKIPKIPKMSDLLETSNMLEMPKMSELSEISDLLETSNMLEMPKMSE | |
| LSEMSDLLETSNMLEIPKIPKMPKVTRMLEISKIPKITKTANNGIVLMNRYDLERVSLLFFKLCDRDYEGKLEIYFKDND | |
| ILGIKSESDVKISKGTHKEHLNTHSSLELSVQGVQLDKSSATLIRSNCTLILTAPKAX | |
| ..............................................................................TT | |
| .T...T.....T.................................................................... | |
| ................................................................................ | |
| .......................................................... | |
|  |  |
| Name: cand007610 | Length: 131 |
| MNSYAAKKSCVDGSFQQVEYSGGFEERYINRIVVRHNTELLPRTARIDSLIPKEYSGVKVSDKKVSLDPEQRHNIRHLLV | |
| ESDTHDIQLSPTDSISSTRNLSPKSPPGSEDFLIFLSEEQLLQTIQPSSSX | |
| ................................................................................ | |
| ...........T.S.SST...............................S. | |
|  |  |
| Name: cand010540 | Length: 591 |
| MKSLSALAIFTLTTSFIYLGESKTGLTCYEPNELSENFQSVTCSDIRSIIGNGGANTFIIDTNSEEIVNCTWIPDKTCTV | |
| LSENKYNNGLTSKCLGFAGFNESEIYIGPHPVNVATSKIAGVLCCSALCADESQNGQSTETKQIVEEISDLFTSKKVTAV | |
| SSSCQNPQELGILDNLSCSQLRDPEVNSKLTYVGNPYYLENKCTFNYDVNCRPFESKNCLGYVINGNHEVWFGIQPVQSI | |
| GPLVQNVLCCSVSCGDQQNFELTNIGNVSNSSQISTPSPNSNNYSDTIVPILPGQSGSQDIPIANNSVVTTVTHPDGIIV | |
| NPETQITEPNPTLSGSENSTAIIKEVQNSEISQSTLNMNSPQTDSQQINSSIQNSSENEIQITTSTTSAPPVISETYSGT | |
| EFTNSVPVPSTNQESNTPIKSFTNTTEASMSSSGSESDIQVVVTTTSTSETFNVVTFPSSNLKNETRPEVQTGNPETQEL | |
| LNSILSCSPPTKFNIPSVTTCEELREWANLSSLIILSHSISQCRWHDEPTCTLSSASCAAYVSTGTDIIFVPGSIPQEGI | |
| SGIQKLSESEQRRLRGKAAEFMPLCCNVVCX | |
| ................................................................................ | |
| ................................................................................ | |
| ................................................................................ | |
| ..............................................T................................. | |
| ...T..T.......................................................TT.TT......S.T.S.T | |
| ..T.......T.....T.....T.T..................TTT.................................. | |
| ................................................................................ | |
| ............................... | |
|  |  |
| Name: cand007370 | Length: 600 |
| MYISQSFFFLVFLQAFSLAAVIIPVEEADALGNNHIVTSIHPSSKILKTDNGQPLNGKSSISNYFNISSMLSSMETLKTS | |
| TRQSLNNITSPTSIFRTNLSTEKSAFTLNSTISRVRSLKSADGLKLTKSISTVKSPFSSTTTPKITIATTGTTTVTTPTT | |
| TTPTTTILTTTTILTTTTVLTTTTILTTTTPTTTILTTTTPTTTTILTTTTPITTTTLTTALTTTLTTTLTTSPTLTTTL | |
| TTSPTLTTSSITSSKTTSPTTTSSKTTTSSKTTTSSKTTTGSKTTTSPTTTSSKTTTSHTTTSSKTTTSPTTSSKTTTSH | |
| TTTSPTTSSKTTTSHTTSPTTTSSKTTTSSKTTTSPTTTSSKTTTSHTTSPTTTTSPTTTSSKTTTSPTTSSKTTTSPIT | |
| TTSSKATTSLTTTTSPTTTMRSVTIRSTTMFTTKINRGFHKGKCESKFTMWYENGIPLSSIQCSNYSVVYMYNEPPFYVS | |
| KPIRQIKVFSNGSITVNNVFLPISGISKRFPSMQYGFMLGYTNIGVSILLNNSYYTLSANGTDFYFGSESDSLFVLSNED | |
| GIFHDGVRLYPTNSKASSASAKSVFLSIVVTIFITTFILX | |
| ................................................................................ | |
| ...........................................................TTT...T..TT.TTT.TT.TT | |
| TT.TTT..TTTT..TTTT..TTTT..TTTT.TTT..TTTT.TTTT..TTTT..TTTT.TT..TTT.TTT.TTS.T.TTT. | |
| TTS.T.TTSS.TSS.TTS.TTTSS.TTTSS.TTTSS.TTT.S.TTTS.TTTSS.TTTS.TTTSS.TTTS.TTSS.TTTS. | |
| TTTS.TTSS.TTTS.TTS.TTTSS.TTTSS.TTTS.TTTSS.TTTS.TTS.TTTTS.TTTSS.TTTS.TTSS.TTTS..T | |
| TTSS..TTS.TTTTS.TTT............................................................. | |
| ................................................................................ | |
| ........................................ | |
|  |  |
| Name: cand015210 | Length: 226 |
| MVYLKSICTSFCLLSSMSSLHAGTFRSLVTEVISESPTITTTVQPYSVELATTNPIKKSKDTELLSEVYSQILNSTNDER | |
| TLLTSSFSFWDHVKANINSILNKLRSDIHNGSKEFEEFRNNTGHFFEEAGSNIEYIISRASYFMNRLGYNMDNFVNYTSP | |
| KVLDHTKELVHEGISLFGGVGDVSKVGHETVPVIETITKEIGGAVDGTKSELSDIKSQTEEIACDX | |
| .............................T.......T.TTT.........TT........................... | |
| ................................................................................ | |
| .................................................................. | |
|  |  |
| Name: cand019690 | Length: 248 |
| MKSARYNSSSTYTRRSSKSSTNSDITNQSNISRSSGYSSTSRKSNRTRRTNNSNKNGTNSAGSNSSLNSSLSNDSDSVSS | |
| VETSISKGTQVSSSSKSSKSSKSSKSTKLSNNSKLSNKVIICRDDKEIKSGCESTHPTQRNKVSINKEIQDLQLSKKKGS | |
| LKMKSAKNEQVKQIPSRNCSFDGHMLTHGNIFFVNCHNYPTGQINIVNQWGSVIVEPQIRCCCNLPNLCLFNCIYDDNPM | |
| SLTILNYX |  |
| ..........T.........T....T.....................................................S | |
| ..TS.S..T..SSSS.SS.............................................................. | |
| ................................................................................ | |
| ........ |  |
|  |  |
| Name: cand015870 | Length: 123 |
| MDVSDLSTLCVISIIVLVLVSWIMSTHLKHEETIPKRRIKKKIVKQKPAETNEEKTEILTTHRPRARSAKAEVIGQNKTS | |
| TKSNATDDPIRTRRNASLKKSNATEEAPVTKKRVTRSSSKKLX | |
| ...........................................................TT.................T. | |
| ...........T...........T.....T....T.SSS.... | |
|  |  |
| Name: cand015230 | Length: 441 |
| MLKKIYFINIIFLIVKYVSCHIENYLDISPLYIDDYSHIIQLHSGATGASTDMPIKSILTDIHNSYDYTNKKLEVADSNI | |
| RSDLDLSLQACSLGTKDKHVTPMTEISCNERLSDTDKEILNIYSNHKVKADPSLEGHFAVSGKINPSSVNKMISDTNPAE | |
| VGFQGSDNVIPIQLNSFRSSNVGVHSNIGINQPCYEGTENLMVLSSAPTNKLLSHSSNVHSDLPIVQSSKLSSALSDSSS | |
| GHSVGLPSDDHVIPYTDISPNLNSNSYLKQYVDLLSTRHLDTKDSSSYTNSIPLGLSSELPVDTKASSQSNTQTTPPDNS | |
| HISSNTAALSPEDIAKIASSVGSSLLLAIGGVVGGGAAYRLNYMKRKEDISNKLISKLKSQSIGNITEVILEESNLPGKE | |
| DIVGIVNTASKVKRSLDSKPGFNRMVYRRRHTKFRVNKQDX | |
| ................................................................................ | |
| ................................................................................ | |
| ................................................................................ | |
| ...............................................................T.......T.TT..... | |
| .....T.......................................................................... | |
| ......................................... | |
|  |  |
| Name: cand020400 | Length: 305 |
| MKTKTLVFALIFGALMPTYILTKTSEIFYLIEPADIPDKKEQNKEFSELFPTAFDEIPRAEIMYSEDTAKPSSGFLPVLF | |
| TSSLAKNNILDIGGSNRELMPNSFENIFISMEENMNLPVFPSKEYKPIINTIITNEKSKDLEFGVKSDMRISQSSSKPLL | |
| LFSREPSSTYPENTYLPTSMDEINLEDKHTPLITNSYNEFKYIPYGNKKEKLRRARRLGLQIPTGKPGPSPFEPRSTKEI | |
| IAESNIDTTSPNLKNNATIINSGFGIVSITNDLLNEEVGVSSKSHIRTSLTFIKEQTNSSATNSX | |
| ................................................................................ | |
| ................................................................................ | |
| ...............................................................T...........ST... | |
| .............................................................T.S. | |
|  |  |
| Name: cand018310 | Length: 182 |
| MADTQNQVEIPKLFLSSTSTKKSDRGNSEESKDLSRKLKRTLGSSGTSSPEPKLKSPGVDDEDELSDTSLDLPDEYFKKA | |
| VMLNINPFDPFPEIDTTAFATGVGKSGDKDIDLTGKLERKKVNTKLSGGDPVESETEEDSSHGRDVLLKLPSNLEVPNMK | |
| SSSPKKSDKSKRQKKTPKKKDX | |
| ...T....................................T.....T........S........................ | |
| ................................................................................ | |
| ...............T...... |  |
|  |  |
| Name: cand020150 | Length: 232 |
| MHINLLWFLCITILPGAILCIPTILPHAISSSLGHLASNAINKHANTIAHSAVLSHTANIISSHAAKAIVVHASHTPVQH | |
| IQNPTSIHSNNIIQSHSTNVAHSANVANFGSHLVPSTHQNHLAASSSHGSISHGTGDSTKFTLIHHSLQYNAHHPLSYTF | |
| AESTSGKGISTLDKTPSTSTVPNIKPDSILAANIISVVLTGIQIAISVTGISFTIYHTTKSRRSRKPYYVRX | |
| ................................................................................ | |
| ................................................................................ | |
| ..........T...T.ST.T.................................................... | |
|  |  |
| Name: cand020430 | Length: 147 |
| MNFYILTFLYLFFLECNALVGPLEPPPNGAPIVSDLRSSVEKMRRLLVHAPDKNSQEKANAFISLDELEKAVEKVGFLAS | |
| KGKTSSEVVLLESNEQPEALTETTPEEATEAIKQLTRQIWKATDDILRAYTGIGGLVGALPPQTISX | |
| ................................................................................ | |
| ....................T.TT....T..................................T.S. | |
|  |  |
| Name: cand016440 | Length: 89 |
| MGCSSSKPVAKSDSKPPNDIERQRILAEKKAELAKAMKKPNKDISKPLMETEEIDKEGDSKVNEPVLLTKPESTSEGYTT | |
| ETNKPAEEX |  |
| ...SSS..............................................................T...STS...TT | |
| .T....... |  |
|  |  |
| Name: cand013930 | Length: 143 |
| MHMQGLCVKCVNRVQSYNLPCVTILPPSQLRKFLSPLYIIKLYLSLKENESKDSVQLRYYSNGISSNFSAGPFSSSLGST | |
| SFRHVDFNGGGRGRGRNSGNSSGNSSGNSSGNSSGNSNSNPSNGNSSGNSSGNSSGNSSGNSS | |
| ......................T......................................................... | |
| ................................S...S.............S..SS..SS..SS | |
|  |  |
| Name: cand024530 | Length: 168 |
| MYRPQVIYWLNLLILLFSIYYVLANQVYISVDSTGKIENLVNNIPDKKDLLTSNLLKATNLMKIKSNEEFENCSCVVGNS | |
| CLTFGDCYGAAAATVIGILIILSTIGIIVQHFAGIPDEFLTTHHPAIVDSARIPCNCLCPIQIREPAHPDMKTSVNVIDS | |
| PTVSSESX |  |
| ................................................................................ | |
| ........................................................................T......S | |
| .T.SS.S. |  |
|  |  |
| Name: cand036430 | Length: 474 |
| MKAFLIFSVISSLLLINASSISSFSSQVGQVYRESGDRSHDDQNNKTRTMGGLPRSSNVRKPTPSLSNSSKVDLRNMNFS | |
| LPLLNSTIVLPKPISKRTTTTTTSKTISENKSKSSSLPITELIAREQSLFYPIGNGISFFPYKPGYFNIPPTTTTTVTTT | |
| TTTTDTTPTTTTESDGTSFFPYKPGYFNIPPTTTTTTTESTTTTTTTESTTTTTELPTTTTTTTESTTTTTTESTTTTTE | |
| STTTESTTTTTDSIPEISDDPLGSDSSKLQSLLYQTGEGILRVPMKIGPGKGQITTTAITSSDSVDLSKGMGQQPILYPI | |
| GSNSTSGVPLLSGKLGSKRLKTIITPTTVSTTALAETTIQQRIENSNDGDKSGMPYSLYPIYGEGMKLFPMKPVYGSPPI | |
| TTTTTTTTTSNIRTSTVTYTYGANGAVAKPHNSIKVRTSHDVKEYEVRSREVGSPSQYNSYTILPVNGSQVVPX | |
| ................................................................................ | |
| ..................TTTTTS.T.............................................TTTTT.TTT | |
| TTTT.TT.TTTT.S..TS.............TTTTTTT.STTTTTTT.STTTTT...TTTTTTT.STTTTTT.STTTTT. | |
| STTT.STTTTT.S................................................................... | |
| ................................................................................ | |
| TTTTTTTTT....T.T.T........................................................ | |
|  |  |
| Name: cand033430 | Length: 266 |
| MRLLYLVFLLIIPIVTNSHQDVLFMHEDVVDHNHMDNLLGNIQNHYQDIQHEHQYDHNLYDISNTHAQMGLNALQAVKLH | |
| NQFENNNYNRHGTGEHYSTNPDMNAIQNKKWQTGKVNFIFGPDLPQPPPLTTHEPMITGVTSSEHHDTTIPSHSLGTTIH | |
| TDLHTTNKINHQEETTINQRGHKYTTISKSNKPDNEHTTFTTEAVTKPTTHHTEVSTSPKAHTTEVTTRSITHSTETTKL | |
| TTRYTETTTKPTTIHTETTTKPTTHS | |
| ................................................................................ | |
| ..................................................TT.....T..TSS....TT........... | |
| .....................................TT.TT...T..TT..T..STS....TT..TT.S.T.ST.TT.. | |
| TT..T.TTT..TT..T.TTT..TT.S | |
|  |  |
| Name: cand031810 | Length: 283 |
| MLDSSNKENSVLAQIGRKSSLYKFIKKLKEPTNQLKSATPSETSESEELNSTISSPSIYNSLNFSPIHQEPEGYEKLGSA | |
| ITDSSKKFDTDKSRTVSQKKTANQATFIESKYLQPNSTSKKPISKSLEKLYASPKNYRNIYPMFSPYQACETLINCNLTS | |
| LCPRTMCICMYNICPNQNYSTSSEVKPWSDQLGCKTCFGRTQQNMLMVARNKVQCKYHCQGSSQAMPYRLPPAIRFNFCN | |
| SSNGSLNCYNSSNSFSTCCNICNSCRFCRLNSLNNCCIVYNQX | |
| ......................................T.S.TS.S....ST............................ | |
| ................................................................................ | |
| ................................................................................ | |
| ........................................... | |
|  |  |
| Name: cand025220 | Length: 184 |
| MIDPASAFCVGVYFLGVGVVSSVLYIPISLMQAMESYRLNEVNKDDTQDLDQLSIDNTYCVGKTSGLLKRKVLCSVVSNK | |
| RKGDILEDQIKPYVLRYPLVLVEDHLGQCSKGNFSDKDAIEDSISESTCLSTSSVESASSCSAMESSPPITLSSSDFVMD | |
| DLSLALDIATIPEDPRNVAWFRMX | |
| ................................................................................ | |
| ...............................................T...TSS..S.SS.S...S....T......... | |
| ........................ |  |
|  |  |
| Name: cand021870 | Length: 503 |
| MLLFLTNIYLISVYGSIFIYRVNCQNFRTYVPLNNSSYNIPWNSKDDISAIGLNNLTLTKANNSLGLLDFDSTDAINMTA | |
| YMMTHYTNQSLSNNFVNNLNNENATFVLPPIISLNNTINSSSSSSSSSSSSSSSSSSSSSSSSSSSSSDSESEGDLKYPK | |
| NKTNYTNIKFDGLSEGLDVKNKTLFIPNVDSQNLRKLSQILIEVNEDIPSTLNVNPQENITIDNNLTTILNITDTLTSNS | |
| FDNIDTNNSHKVKELYPILLNSNITETSKNLSNDTLIIANEVLNNTIGNFNHDNNITSDTYILLSKSNDIVTEPLDPMSV | |
| VLLPINITDKNKTILPQNINSNDVSDIKTCNMSTILNNSFVNTASDPLKASNFIKNDSIVKQDKLYDELLKNTSFNSSFL | |
| KNKIKSINTLEIIPSKNSTYLDLNNTSKGSCPNGICKSDGEDDSESDDEVELDENGKPIIKFKQHSIEIDEIIKTANAMK | |
| QEPYLASLLLSCTFLISLFVFLX | |
| ................................................................................ | |
| ....................................T..SSSSSSSSSSSSSSSSSSSSSSSSSSSSS............ | |
| ................................................................................ | |
| ................................................................................ | |
| ................................................................................ | |
| ................................................................................ | |
| ....................... |  |
|  |  |
| Name: cand029820 | Length: 98 |
| MTSDKKKGTCGVAFETILNGLGLLGNQFRTALIATKEVVKDSVYPLKENCIRRYYEVNPAWHDIGSKTSVPNQNAVLFAD | |
| SGASETIDTQSSIRSTLX | |
| .T.............................................................................. | |
| ........T..S..ST.. |  |
|  |  |
| Name: cand037300 | Length: 269 |
| MSATINKENFLILSEKHKYYNEYAGIGTPDISTSPGTPSPDSLEVSTMSSLWTYKTKLDRIENTIDYIKREYLSEVSSLQ | |
| DRYNLATLDKMEANIKELRKKYNLIFSKYEEHQINKIIQITKKSQVSLINIQNLVDKITRRSQETEIEQNFKCTTELSKL | |
| ELEIELETLKLRLDRDLKFNRYLTVCNDKLREKYKELSKIYDTLEMYHESKCNYLRKESNEIYRTRNSLLNRDLLDNQNI | |
| IDLLVFENELQAEILGYFKNLKLIFSQSX | |
| ...........................T....TS..T.S..S....T................................. | |
| ................................................................................ | |
| ................................................................................ | |
| ............................. |  |
|  |  |
| Name: cand027340 | Length: 436 |
| MSSTTVVKPRVPTRARGERVYKPGGKKEWRVVFWTHESNKPSRRQCSFSEAKYGKEAASLLSRAVLDYIDVKGVIPDDLH | |
| DPPILDPAKKELIDYYSALHETRKASQKKIRQKSKYKQSQEESIVPLYTLTQSASLTNISSKKKLSLLQPVSSLSDTLVY | |
| LMNSKDNNNMSNKSEIEGIKNNGDNSLSTTSIFSTTSSTSSVSNSSDKCSLSPSVSPLSIATNHIIRGSNTDSDIGLADN | |
| IYNPGILTSFGNLGNCNIPNAEHIGSQFPITKGPSYIDNNTNESQNRNFTTVTNFLLNNLSKNVLLSGNMISPLNSLINQ | |
| QYPTVLPPPLPDILSNNQLPLNSDLQLFINQTFQQLYQNQFSLINMSHFQYIAINNPPIVSIQGIDTLKIADQQSYIDPF | |
| SKLRSPATNSDMELTKSNVNPSLTKETSSDSPKPVX | |
| .SSTT........................................................................... | |
| ................................................................................ | |
| ......................................TSS.S.SS.................................. | |
| ................................................................................ | |
| ...T............................................................................ | |
| .......................T..TSS.S..... | |
|  |  |
| Name: cand023130 | Length: 157 |
| MTWPFSSAEKKNISSPIDEFVPPPPGGSPFAPPPEIPHSLKHIKDHSFFHKEFKGFSAPPGRSIDSETRTYEQRFPNSKK | |
| SIFDLEFSPRTRACLENIKMGMKMGASVGGIFGALTGVYAAAKHRNLLAIPISIAGGAVSFGFFLGCGMIVRCESKX | |
| .T....S......SS............S.......................................T.T.......... | |
| ............................................................................. | |
|  |  |
| Name: cand022470 | Length: 551 |
| MNTIYIWIFLYLPLLWMCVVSLIDEQLSLIHDDLINSNILYNYDHLNNDPEIQQENILPNIFENGINLVDNIPNAVHKSE | |
| ILTIPFSNSHIEVPSSIHITYNEKLINSHSNDNSEFKDHLLNLLPHLPNQPTNSNGFPNPSNTEPDSSGGTDHNRDSDND | |
| FPNPSGGTTSHTTATPITTFIHITSSSNPSHSVSTTYTTNPITTSIPSTTYTTNPIITSTHITSSSNPSHSEGTTYTTTN | |
| PITTTSTHITNPSHSEGTTYTTTNPITTTTSTHITSSSNPSHSEGTTSTTANPITTSIPSTSSSNNENHGNSNSVHTGSS | |
| KPNIGTIIGGTLGGLLLVGGALVGGGLFINNRRIKKERLRNKLRRKKKRRQDLTSASIGDSTIVSQNSVSSSVLPSKRPL | |
| KTSVIDDSDSLVTLEVSHGQTFDGLGSTTLLPLPAIKINSGDEPSSKSVSNKTTKLDDYSQTFGGLGSVTALPLPIIKIN | |
| NGYKPSNKSASKKTRKVNASNINKEIPKGSIHNSTSNANPEPQQLPIFLGNTTRKKVSLSVSDLTGSLSIX | |
| ................................................................................ | |
| ..............................................................T.......T......... | |
| .......TT..TT.T..TT....T.....S.S.STT.TT...TTS..STT.TT....TST..TS....S.S..TT.TTT. | |
| ..TTTST..T..S.S..TT.TTT...TTTTST..TSSS..S.S..TTSTT....TT....T................... | |
| ................................................................................ | |
| ................................................................................ | |
| ....................................................................... | |
|  |  |
| Name: cand021340 | Length: 287 |
| MRNLINIISLLFGLLGLIYALESTAADPKDQVHTPETKIPIPNNNTVPLVKEPQESARITKNQVPISTVFDFPRQYYSAY | |
| TYPIGRHHRHHHHGNFYGVPVAIQPVAIQPVATQPVISKTPVANLTTKEPIIIQNKQPVITNKLPRQTLDKAVDYRAEQI | |
| LLEEPDIRMMVEYPYRGPYVFHPYAYHHYDYDPYFINLSPYNQYPIYRMGYLGHPYSPFYYYRSPYIGGRYSHRFRYLDD | |
| LPPQEGEFIEFPEEEFESFETNMPFEGHSLPEVHRNSTLPTAPLKMX | |
| .................................T..T........T.................................. | |
| ................................T......T.....T.................................. | |
| ................................................................................ | |
| .....................................T..T...... | |
